# scGACL: a generative adversarial network with multi-scale contrastive learning for accurate single-cell RNA sequencing imputation

**DOI:** 10.1093/bib/bbag018

**Published:** 2026-02-03

**Authors:** Yanlin Jiang, Mengyuan Zhao, Jiahui Yan, Jijun Tang, Fei Guo

**Affiliations:** College of Engineering, Southern University of Science and Technology, No. 1088 Xueyuan Avenue, Nanshan District, Shenzhen 518055, Guangdong, China; Faculty of Computer Science and Control Engineering, Shenzhen Institutes of Advanced Technology, Chinese Academy of Sciences, No. 1068 Xueyuan Avenue, Nanshan District, Shenzhen 518055, Guangdong, China; College of Engineering, Southern University of Science and Technology, No. 1088 Xueyuan Avenue, Nanshan District, Shenzhen 518055, Guangdong, China; Faculty of Computer Science and Control Engineering, Shenzhen Institutes of Advanced Technology, Chinese Academy of Sciences, No. 1068 Xueyuan Avenue, Nanshan District, Shenzhen 518055, Guangdong, China; School of Computer Science and Engineering, Central South University, No. 932 South Lushan Road, Changsha 410083, Hunan, China

**Keywords:** single-cell RNA sequencing, data imputation, contrastive learning, generative adversarial networks

## Abstract

Single-cell RNA sequencing is a powerful technology for investigating cell-to-cell heterogeneity, yet its application is often hindered by dropout events, making accurate imputation essential for downstream analyses. Existing imputation methods, however, frequently suffer from the over-smoothing problem, which results in the loss of cell-to-cell heterogeneity in the imputed outcomes and affects downstream analyses. To overcome this limitation, we propose scGACL, a generative adversarial network (GAN) integrated with multi-scale contrastive learning. The GAN architecture facilitates the distribution of the imputed data to approximate that of the real data. To fundamentally address over-smoothing, the model incorporates a multi-scale contrastive learning mechanism: cell-level contrastive learning preserves fine-grained cell-to-cell heterogeneity, while cell-type-level contrastive learning maintains macroscopic biological variation across different cellular groups. These mechanisms function synergistically to ensure accurate imputation and effectively address the over-smoothing challenge. Comprehensive evaluations across diverse simulated and real-world datasets confirm that scGACL consistently outperforms existing methods in accurately recovering gene expression and improving downstream analyses such as cell clustering, gene differential expression analysis, and cell trajectory inference.

## Introduction

Recent advances in single-cell RNA sequencing (scRNA-seq) technology have enabled researchers to investigate cell-to-cell heterogeneity, cell differentiation processes, and complex disease mechanisms [1, 2]. However, scRNA-seq data are highly sparse and contain a large proportion of zeros. Previous studies have shown that these zeros can be divided into two categories: biological zeros, which represent actually unexpressed genes, and non-biological zeros, which result from technical limitations that prevent the detection of expressed genes [3]. These non-biological zeros, commonly known as “dropout events,” severely impact downstream analyses [4]. Therefore, accurately imputing dropout events is a critical task in scRNA-seq data analysis.

To address dropout events, various imputation methods have been proposed, which can be grouped into four categories [5]. The first group performs imputation by aggregating gene expression from similar cells. For example, MAGIC [6] applies data diffusion to share information between similar cells, thereby denoising the gene expression matrix. However, these approaches often excessively homogenize the data, which diminishes the biological differences between cells [7]. The second group employs probabilistic models to fit the scRNA-seq data distribution. For instance, SAVER [8] and VIPER [9] model gene expression using Gamma-Poisson and zero-inflated Poisson (ZIP) mixture distributions, respectively. Their main drawback is reliance on these specific distributional assumptions, which may not be suitable for all datasets. The third group uses matrix factorization, treating imputation as a low-rank matrix completion problem. For example, ALRA [10] utilizes low-rank matrix approximation to impute dropouts while specifically preserving biological zeros. To further improve imputation accuracy, SCRABBLE [11], based on matrix regularization, utilizes matched bulk RNA-seq data as a constraint during imputation. Similarly, scINRB [12] is a network-regularized non-negative matrix factorization model that leverages bulk RNA-seq data to guide imputation. Although incorporating bulk RNA-seq data into the imputation process helps correct expression biases, these methods may become less effective when such data is unavailable [13]. While these first three groups of methods are limited in capturing non-linear features from scRNA-seq data [14], deep learning-based methods are better suited for this task.

In the field of scRNA-seq data imputation, commonly used deep learning models mainly include autoencoders, graph neural networks (GNNs), and generative adversarial networks (GANs). For example, scIGANs [15] employ a convolutional neural network (CNN) by treating cells as “images” and genes as “pixels.” Nonetheless, this approach is ill-suited for scRNA-seq data, as CNNs assume meaningful spatial relationships between adjacent pixels, a property not held by neighboring genes in the “images.”

In addition, scGANCL [5] improves imputation by integrating a bidirectional GAN (BiGAN) with contrastive learning. scGGAN [16] combines graph convolutional networks (GCN) to learn gene relations and GANs for global data distributions. It constructs a gene network from single-cell and bulk genomics, then integrates this with sequencing data into a GCN-based GAN for imputation. For autoencoder-based methods, AutoImpute [17] employs an autoencoder to reconstruct the expression matrix. Deep Count Autoencoder (DCA) [18] extends this approach by incorporating a zero-inflated negative binomial (ZINB) distribution model. Bubble [19] is also based on an autoencoder, and introduces bulk RNA-seq data matched with scRNA-seq data as a constraint to a ensure more accurate estimation of gene expression levels. For GNN-based methods, scGNN [20] employs GNNs to model cell–cell relationships and imputes dropout events.

However, the imputation results of autoencoders and GNNs are often overly smoothed [21, 22], which may reduce cell-to-cell heterogeneity. Specifically, over-smoothing refers to the phenomenon where distinct gene expression data become homogenized during imputation, causing biologically different cells to converge toward similar expression patterns. This occurs in autoencoders due to their information bottleneck, which compresses data through a low-dimensional latent space and forces the model to learn averaged representations at the expense of cell-specific details. In GNNs, the issue originates from the iterative message-passing mechanism, where features are repeatedly aggregated from neighboring cells across layers, causing distinct cellular representations to become homogenized.

Compared to autoencoders and GNNs, GAN-based methods offer the distinct advantage of learning complex data distributions through adversarial training, which is crucial for generating realistic imputed values in sparse single-cell data. Therefore, we adopt a GAN-based architecture for the imputation task. Nonetheless, standard GANs are prone to training instability. To address these limitations, we propose scGACL, a novel imputation model that integrates a GAN with multi-scale contrastive learning. Specifically, our model consists of a generator and a multi-task discriminator. We employ the Variational Deep Embedding (VaDE) model proposed by Jiang *et al.* [23] as the generator, which is a Variational Autoencoder (VAE) integrated with a Gaussian mixture model (GMM). The core innovation is the multi-task discriminator, which comprises three modules. An adversarial discrimination module is adversarially trained against the generator to enhance the realism of the generated data. To address the over-smoothing problem, the other two modules implement a multi-scale contrastive learning strategy: a cell-level contrastive learning module preserves cell-to-cell heterogeneity at a fine-grained scale, while a cell-type-level contrastive learning module maintains biological differences across different cell groups at a macroscopic scale. To address the issue of training instability, we first pre-train the VaDE generator and then jointly train it with the multi-task discriminator.

Through comprehensive experiments on multiple simulated and real-world scRNA-seq datasets, we demonstrate that scGACL consistently outperforms state-of-the-art methods, not only in accurately recovering gene expression but also in enhancing key downstream analyses such as cell clustering, gene differential expression analysis, and cell trajectory inference.

## Materials and methods

**Figure 1:**
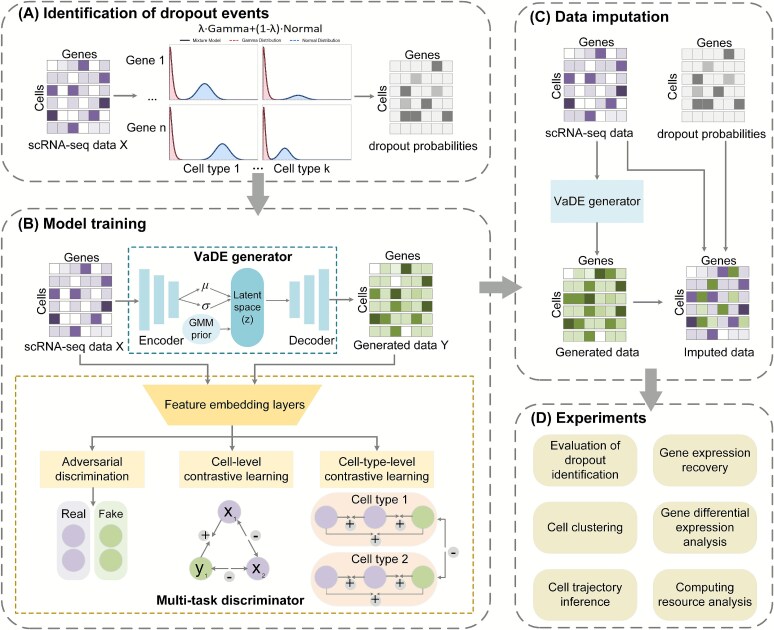
The workflow of the scGACL framework. (A) Identification of dropout events. Dropout events in the scRNA-seq data are identified by computing dropout probabilities using Gamma-Normal mixture distributions. (B) Model training. The scGACL architecture consists of a VaDE generator and a multi-task discriminator. The VaDE generator encodes the observed scRNA-seq data X into a latent space guided by a GMM prior and decodes it to generate new data Y. The multi-task discriminator processes both observed (real) and generated (fake) data through shared feature embedding layers and performs three tasks: (i) adversarial discrimination between real and fake data, (ii) a cell-level contrastive learning module, which treats an observed cell embedding ($x_{1}$) and its corresponding generated cell embedding ($y_{1}$) as a positive pair, while treating all other non-corresponding cell embeddings as negative pairs; and (iii) a cell-type-level contrastive learning module, which treats cells of the same type as positive pairs and cells of different types as negative pairs. The “+” and “–” symbols denote positive and negative sample pairs used in the contrastive learning modules, respectively. (C) Data imputation. The scRNA-seq data to be imputed are fed into the VaDE generator trained in step B to produce generated data. Using the dropout probability matrix obtained in step A to distinguish dropout events from biological zeros, we then use the generated data to impute the dropout events, yielding the final imputed results. (D) Experiments. The performance of scGACL is validated through multiple downstream experiments.

We propose scGACL, a novel scRNA-seq data imputation method that integrates a GAN with multi-scale contrastive learning. The overall workflow of scGACL is illustrated in Fig. 1. In this section, we will detail its main components: the identification of dropout events, the model framework, and the associated loss functions.

### Identification of dropout events

To avoid over-imputation, we impute only the zeros identified as dropout events. Inspired by scImpute [24], we use a Gamma-Normal mixture model to identify dropout events. In the mixture model, the Gamma component represents dropout events and the Normal component represents gene expression levels. Since model parameters vary across cell types, we fit a separate mixture model for each cell subpopulation. If cell type labels are available, we use them to define these subpopulations. Otherwise, we perform K-means clustering to generate pre-clustering labels, which then define the subpopulations. The number of clusters $k$ is determined using the Elbow method and Silhouette coefficient, following the strategy used in AutoClass [25].

We denote the preprocessed expression matrix as $X$ (details of the preprocessing are provided in Supplementary Note S1), where $X_{ij}$ represents the expression of the $j$th gene in the $i$th cell. For each gene $j$, its expression in cell subpopulation $k$ is modeled as a random variable $X_{j}^{(k)}$ with density function:


1 \begin{align*}& \begin{split} f_{X_{j}^{(k)}}(x)&=\lambda_{j}^{(k)}\operatorname{Gamma}\left(x;\alpha_{j}^{(k)},\beta_{j}^{(k)}\right)\\&\quad+\left(1-\lambda_{j}^{(k)}\right)\operatorname{Normal}\left(x;\mu_{j}^{(k)},\sigma_{j}^{(k)}\right) \end{split}\end{align*}

where $\lambda _{j}^{(k)}$ is the dropout rate of gene $j$ in subpopulation $k$, $\alpha _{j}^{(k)}$, $\beta _{j}^{(k)}$ are the shape and rate parameters of Gamma distribution, and $\mu _{j}^{(k)}$, $\sigma _{j}^{(k)}$ are the mean and standard deviation of Normal distribution. These parameters are estimated by the expectation–maximization algorithm, and we denote their estimates as $\hat{\lambda }_{j}^{(k)}$, $\hat{\alpha }_{j}^{(k)}$, $\hat{\beta }_{j}^{(k)}$, $\hat{\mu }_{j}^{(k)}$ and $\hat{\sigma }_{j}^{(k)}$. Therefore, the dropout probability of gene $j$ in cell $i$ belonging to subpopulation $k$ can be estimated as


2 \begin{align*}& \begin{split} d_{ij}=\frac{\hat{\lambda}_{j}^{(k)}\mathrm{Gamma}\left(X_{ij};\hat{\alpha}_{j}^{(k)},\hat{\beta}_{j}^{(k)}\right)}{\hat{\lambda}_{j}^{(k)}\mathrm{Gamma}\left(X_{ij};\hat{\alpha}_{j}^{(k)},\hat{\beta}_{j}^{(k)}\right)+\left(1-\hat{\lambda}_{j}^{(k)}\right)\mathrm{Normal}\left(X_{ij};\hat{\mu}_{j}^{(k)},\hat{\sigma}_{j}^{(k)}\right)}. \end{split}\end{align*}

Finally, if the dropout probability $d_{ij}$ is greater than a predefined threshold $\rho $ (default value: 0.5), the zero expression value of gene $j$ in cell i is identified as a dropout event.

### Model framework

scGACL consists of two main components: a VaDE generator and a multi-task discriminator.

#### VaDE generator

Conventional VAEs use a standard normal prior, which is often too simple to capture complex data structures. Therefore, we employ VaDE [23], a VAE variant that replaces this simple prior with a more flexible GMM, enabling more robust modeling of the latent space [26]. The input to VaDE is the preprocessed scRNA-seq data. In the forward pass, an input sample $x$ is first mapped into the latent space by the encoder network, which outputs the parameters of the posterior distribution $q({z},k|{x})$, where $k$ is the GMM component index. From this distribution, a latent code $z$ is sampled using the reparameterization trick. The decoder then generates a reconstructed sample from $z$. This reconstructed sample is treated as the generated sample (fake sample) and used for both generative adversarial training and multi-scale contrastive learning, paired with the observed samples (real samples). Finally, VaDE is trained by maximizing the evidence lower bound (ELBO):


3 \begin{align*}& {\mathcal{L}_{\mathrm{ELBO}}}=E_{q({z},k|{x})}[\log p({x}|{z})]-D_{KL}(q({z},k|{x})||p({z},k)).\end{align*}

The first term in Equation (3) is the reconstruction term, which ensures that the latent code $z$ retains sufficient information to reconstruct the input sample $x$. The second term is the Kullback-Leibler divergence from the Mixture-of-Gaussians (MoG) prior $p(z, k)$ to the variational posterior $q(z, k|x)$, which regularizes the latent code $z$ to lie on a MoG manifold [23]. The detailed derivation of the ELBO is provided in Supplementary Note S2.

#### Multi-task discriminator

While VaDE is effective for modeling data distributions, its VAE-based structure may lead to overly smoothed outputs. To address this, we introduce a multi-task discriminator, ${D}$, which performs both generative adversarial training and multi-scale contrastive learning. Within ${D}$, observed and generated samples first pass through a feature embedding module (${f_{\mathrm{e}}}$) to yield latent embeddings. These embeddings are then input into three modules: the adversarial discrimination module, the cell-level contrastive learning module, and the cell-type-level contrastive learning module.

**Adversarial discrimination module.** We use the adversarial discrimination module ($H_{\mathrm{adv}}$) with the VaDE generator for generative adversarial training. This module takes the latent embeddings of real and fake samples as input and outputs the probability that an embedding comes from a real sample. During training, the generator aims to produce synthetic samples that fool the adversarial discrimination module, while the module improves its ability to distinguish fake samples. Through this adversarial process, the generator learns to generate more realistic data. For the discriminator, the adversarial loss is:


4 \begin{align*}& \begin{split} \mathcal{L}_{\mathrm{Dis}}&=-\mathbb{E}_{{x}\sim\mathrm{p}_{\mathrm{data}}(x)}[\mathrm{log}{H}_{\mathrm{adv}}({f}_{\mathrm{e}}({x}))]\\&\quad-\mathbb{E}_{{x}\sim\mathrm{p}_{\mathrm{data}}(x)}[\mathrm{log}(1-{H}_{\mathrm{adv}}({f}_{\mathrm{e}}(\mathrm{VaDE}({x}))))] \end{split}\end{align*}

where $\mathrm{p}_{\mathrm{data}}$ denotes the distribution of real data. For the generator, the adversarial loss is:


5 \begin{align*}& \mathcal{L}_{\mathrm{G}}=\mathbb{E}_{{x\sim \mathrm{p}_{\mathrm{data}}(x)}}[\log(1-{H}_{\mathrm{adv}}({f_{\mathrm{e}}(\mathrm{VaDE}(x)))})]\end{align*}

**Cell-level contrastive learning module.** To mitigate over-smoothing, we add the cell-level contrastive learning module ($H_{\mathrm{cell}}$) to the multi-task discriminator. We consider a mini-batch of $N$ observed cells. For each observed cell $x_{i}$ ($i=1, \dots , N$), the VaDE generator produces a corresponding generated cell $\hat{x}_{i}$. The combined $2N$ cells ($N$ observed and $N$ generated) are then passed through the feature embedding module ${f_{\mathrm{e}}}$, and subsequently fed into the cell-level contrastive learning module $H_{\mathrm{cell}}$. For observed cell $x_{i}$, it forms a positive pair with cell $\hat{x}_{i}$, and $2N-2$ negative pairs with the remaining $2N-2$ cells. In the latent space, this positive pair is pulled closer, encouraging the model to learn a consistent latent representation for each cell and its generated counterpart. Simultaneously, negative pairs are pushed apart to enforce separation between distinct cells, which preserves cell-to-cell heterogeneity and directly alleviates the over-smoothing problem. Based on the SimCLR contrastive loss from Chen *et al.* [27], the cell-level contrastive loss for the positive pair $(x_{i}, \hat{x}_{i})$ is formulated as:


6 \begin{align*}& \ell(x_{i}, \hat{x}_{i}) = -\log \frac{\exp(\operatorname{sim}(t_{i}, \hat{t}_{i}) / \tau)} {\sum_{k=1}^{2N} \mathbf{1}_{[k \neq i]} \exp(\operatorname{sim}(t_{i}, t_{k}) / \tau)}\end{align*}

where $\mathrm{t_{i}=}H_{\mathrm{cell}}({f_{\mathrm{e}}(x_{i})})$, $\mathrm{\hat{t}_{i}=}H_{\mathrm{cell}}({f_{\mathrm{e}}(\hat{x}_{i})})$, $\mathrm{t_{k}=}H_{\mathrm{cell}}({f_{\mathrm{e}}(x_{k})})$, $\tau $ denotes a temperature parameter and $\mathbf{1}_{[k \neq i]}$ is an indicator function evaluating to 1 if $k \neq i$. The similarity between two cells is measured using the following cosine similarity function:


7 \begin{align*}& \operatorname{sim}(t_{i},t_{j})=\frac{t_{i}^{T}t_{j}}{\|t_{i}\|\|t_{j}\|}.\end{align*}

Finally, the cell-level contrastive learning loss is:


8 \begin{align*}& \mathcal{L}_{cell}=\frac{1}{2{N}}\Sigma_{\mathrm{i}=1}^{{N}}\left[\ell({x}_{{i}},\hat{x}_{{i}})+\ell(\hat{x}_{{i}},{x}_{{i}})\right].\end{align*}

**Cell-type-level contrastive learning module.** Although cell-level contrastive learning is effective at preserving cell-to-cell heterogeneity, it acts as a local constraint that focuses only on single-cell differences and fails to learn the global structure of gene expression data, where cells of the same cell type should cluster and those of different cell types should separate in the latent space. To address this, we propose a cell-type-level contrastive learning module, $H_{\mathrm{type}}$, that leverages cell type labels to enforce this global structure.

We input $N$ observed cells into the VaDE generator to obtain $N$ generated cells. Since the generated cells are derived from the observed cells, they inherit the same cell type labels. All $2N$ cells are passed through the feature embedding module and then input into $H_{\mathrm{type}}$. In this module, positive pairs consist of cells of the same cell type, while negative pairs consist of cells of different types. We adopt the supervised contrastive loss proposed by Khosla *et al.* [28] as the optimization objective. The cell-type-level contrastive loss function is as follows:


9 \begin{align*}& \mathcal{L}_{\mathrm{type}}=\sum_{{i\in I}}\frac{-1}{|{P(i)}|}\Sigma_{{p\in P(i)}}\log\frac{\exp\left(\operatorname{sim}({v_{i},v_{p}})/\tau\right)}{\sum_{\mathrm{a\in A(i)}}\exp\left(\operatorname{sim}({v_{i},v_{a}})/\tau\right)}\end{align*}

where ${v}_{i}=H_{\mathrm{type}}({f}_{\mathrm{e}}(x_{i}))$, and similarly for ${v}_{p}$ and ${v}_{a}$. ${P(i)}$ denotes the set of samples that share the same cell type as cell $i$, ${A(i)}$ denotes the set of all samples except cell $i$, and $\tau $ denotes a temperature parameter. The $\mathrm{sim(\cdot )}$ function refers to the cosine similarity defined in Equation (7).

By explicitly pulling cells of the same type closer while pushing those of different types apart, this module forces the model to learn the desired global structure in the latent space. This preserves macroscopic biological variation between cell types, further alleviating the over-smoothing problem.

### Loss functions

The training process of scGACL contains two stages. In the first stage, the VaDE generator is pre-trained using the ELBO loss in Equation (3):


10 \begin{align*}& \mathcal{L}_{\mathrm{VaDE}}^{(1)}=-{\mathcal{L}_{\mathrm{ELBO}}}.\end{align*}

The pre-training stage enables the generator to produce realistic samples early in adversarial training, thereby preventing the vanishing gradient problem.

In the second stage, the VaDE generator and the multi-task discriminator are jointly trained. The loss of the VaDE generator is:


11 \begin{align*}& \mathcal{L}_{\mathrm{VaDE}}^{(2)}=-{\mathcal{L}_{\mathrm{ELBO}}}+\lambda_{\mathrm{adv}}\mathcal{L}_{\mathrm{G}}+\lambda_{\mathrm{cell}}\mathcal{L}_{\mathrm{cell}}+\lambda_{\mathrm{type}}\mathcal{L}_{\mathrm{type}}\end{align*}

where $\lambda _{\mathrm{adv}}$, $\lambda _{\mathrm{cell}}$, and $\lambda _{\mathrm{type}}$ are the weights for the adversarial loss, cell-level contrastive loss, and cell-type-level contrastive loss, respectively. They are all hyperparameters, and their default values are set to 1. The loss of the multi-task discriminator is:


12 \begin{align*}& \mathcal{L}_{\mathrm{D}}=\lambda_{\mathrm{adv}}\mathcal{L}_{\mathrm{Dis}}+\lambda_{\mathrm{cell}}\mathcal{L}_{\mathrm{cell}}+\lambda_{\mathrm{type}}\mathcal{L}_{\mathrm{type}}\end{align*}

### Data imputation process

After identifying dropout events and training the scGACL model, the VaDE generator takes the expression matrix as input and outputs a reconstructed matrix. This output is used to impute the zeros identified as dropout events, resulting in the final imputed matrix.

## Results

To comprehensively evaluate scGACL, we use eight simulated and seven real-world scRNA-seq datasets. The simulated datasets are generated using Splatter [29] with zero rates ranging from 42% to 91% (simulation details in Supplementary Note S3). The real-world datasets cover human and mouse samples, multiple sequencing technologies (e.g. 10x Genomics, CEL-seq2, Drop-seq), and diverse disease conditions, and are used to evaluate gene expression recovery and downstream analyses (see Supplementary Table S1 for dataset details).

We evaluate scGACL using a comprehensive set of metrics. For dropout identification, we use accuracy, F1 score, false positive rate (FPR), and precision. For gene expression recovery, we use root mean square error (RMSE), Pearson correlation coefficient (PCC), and cosine similarity. For downstream analyses, we evaluate: (i) clustering with Adjusted Rand Index (ARI) and Normalized Mutual Information (NMI); (ii) differential expression analysis with accuracy, F1 score, and Jaccard index; and (iii) trajectory inference with Pseudo-temporal ordering score (POS) and Kendall’s rank correlation score (KOR) [30] (see Supplementary Note S4 for metric definitions and calculations).

For benchmarking, we compare scGACL against 10 imputation methods: scIGANs, AGImpute [31], DCA, scVI [32], scGCL [33], scGNN, SAVER, scImpute, ALRA, and MAGIC.

### Evaluation of dropout identification

To evaluate the performance of scGACL in identifying dropout events, we use eight simulated scRNA-seq datasets. We first generate eight dropout-free datasets (ground truth) with a zero expression rate of 27% using Splatter. We then introduce dropout events into these datasets to create eight sparse datasets (Simulated 1–8) with zero expression rates ranging from 42% to 91%. Model performance is evaluated using classification metrics that compare predicted with actual dropout locations, including F1 score, accuracy, precision, and FPR.

**Figure 2:**
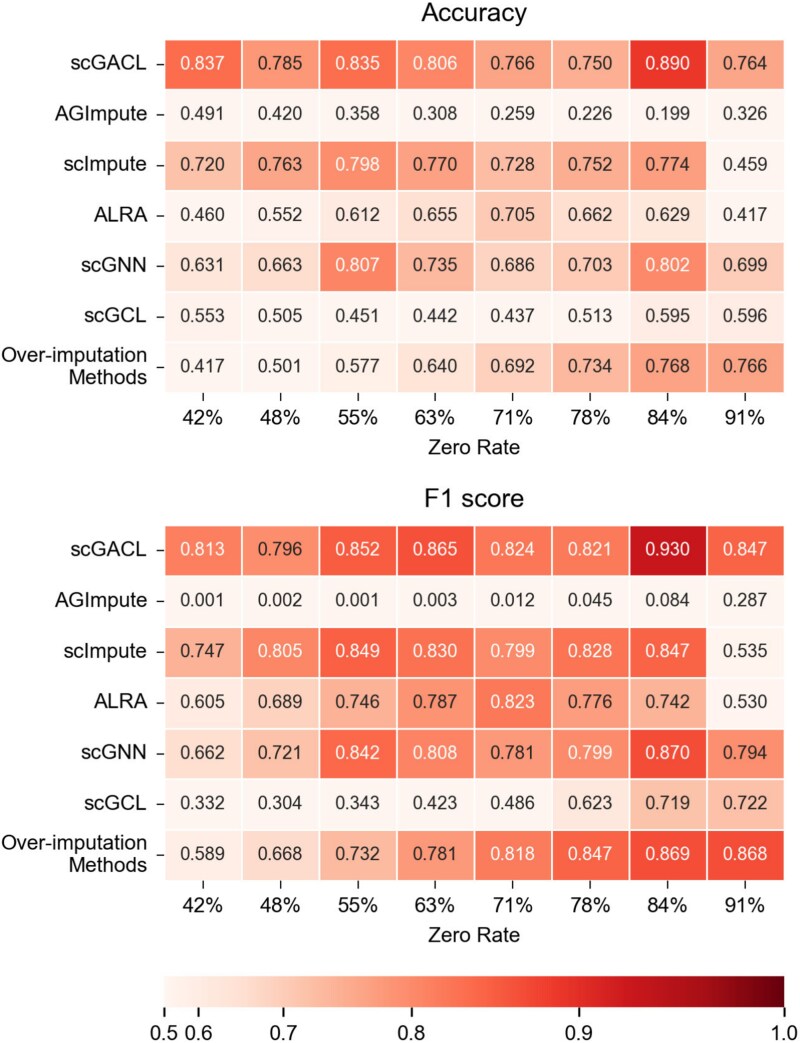
Heatmaps show dropout identification performance (Accuracy and F1 score) across eight simulated datasets, with rows representing imputation methods and columns indicating the zero rates of simulated datasets, where the “Over-imputation Methods” (scIGANs, SAVER, DCA, scVI, and MAGIC) impute all zeros uniformly without distinguishing dropout events from biological zeros, resulting in identical performance metrics.

As shown in Fig. 2, scGACL demonstrates optimal performance in both F1 score and accuracy across nearly all simulated datasets, confirming its exceptional capability in identifying dropout events. Furthermore, Supplementary Fig. S1 shows that scGACL maintains high precision and low FPR. This indicates that scGACL rarely misclassifies true biological zeros as dropout events, a characteristic crucial for avoiding over-imputation and preserving original biological signals. This precise identification stems from the use of Gamma-Normal mixture models. These models effectively fit scRNA-seq data and estimate a dropout probability matrix, thereby accurately distinguishing between biological zeros and dropouts. AGImpute employs mixture models of ZIP, Gaussian, and ZINB distributions to identify dropout events. However, it incorrectly classifies numerous true dropout events as biological zeros, leading to insufficient imputation and consequently the lowest accuracy and F1 score among all methods. The FPR of ALRA exceeds 0.9 on four simulated datasets, suggesting a tendency towards over-imputation. Similarly, methods such as scIGANs, SAVER, DCA, scVI, and MAGIC lack the mechanism to distinguish between biological zeros and technical dropouts. By imputing all zero values without discrimination, they achieve a FPR of 1.0, demonstrating over-imputation as all true biological zeros are erroneously modified. In summary, scGACL achieves superior performance on comprehensive metrics such as accuracy and F1 score by effectively identifying true dropouts while minimizing over-imputation, thereby establishing its key advantage over existing methods.

### Gene expression data recovery

**Figure 3:**
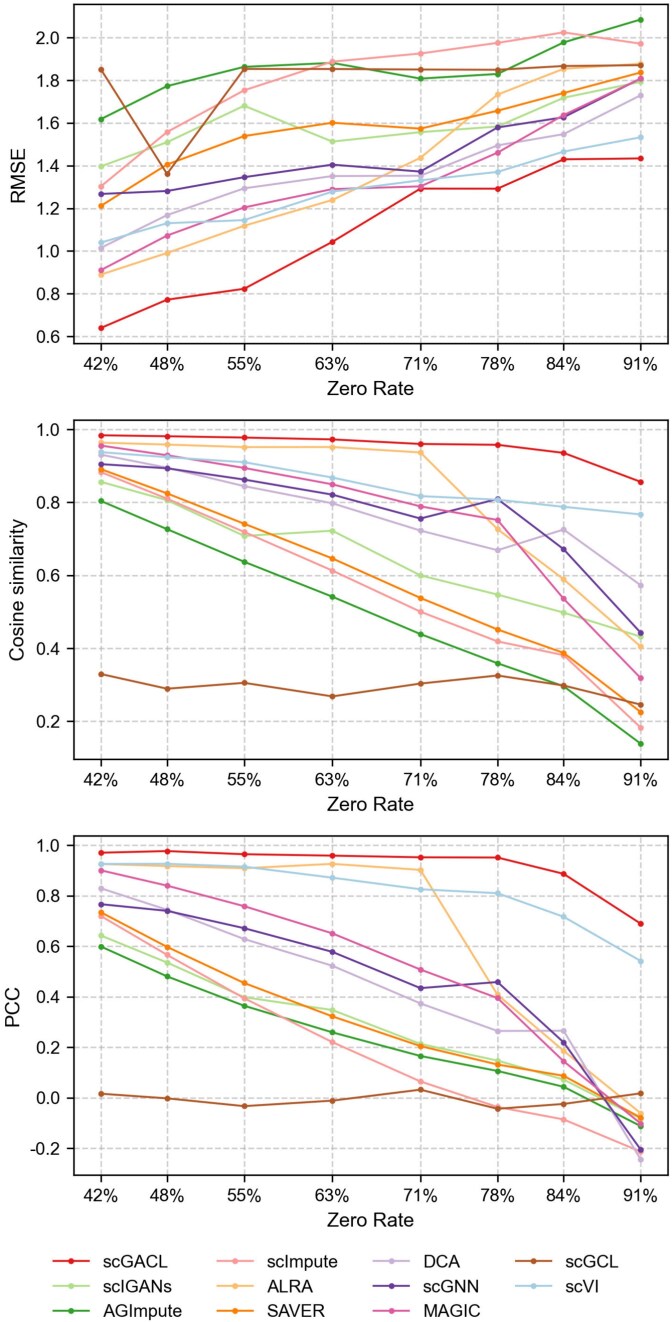
The RMSE, cosine similarity, and PCC between the imputed results and the ground truth on eight simulated scRNA-seq datasets, with the x-axis representing the zero rates of the simulated datasets and the y-axis representing the metric values of the imputation methods.

A robust imputation method should accurately recover gene expression, bringing the imputed values closer to the ground truth without introducing significant errors. To evaluate whether scGACL achieves this goal, we benchmark it against ten baseline methods on eight simulated datasets (Simulated 1–8). We then quantify the imputation accuracy by calculating the RMSE, cosine similarity, and PCC between the imputed results and the ground truth. A comparison of these metrics across all methods is shown in Fig. 3. As the zero rate of datasets increases, most methods exhibit higher RMSE along with lower PCC and cosine similarity, highlighting the challenge of imputing high-sparsity data. Among all methods, scGACL achieves the lowest RMSE, highest PCC, and highest cosine similarity across all simulated datasets, demonstrating its superior accuracy and robustness. In contrast, scGCL exhibits PCC values near zero across all datasets, suggesting that it does not effectively recover meaningful gene expression patterns. Additionally, AGImpute also demonstrates limited performance, with its cosine similarity only surpassing scGCL, ranking as the second-lowest among all methods evaluated.

**Figure 4:**
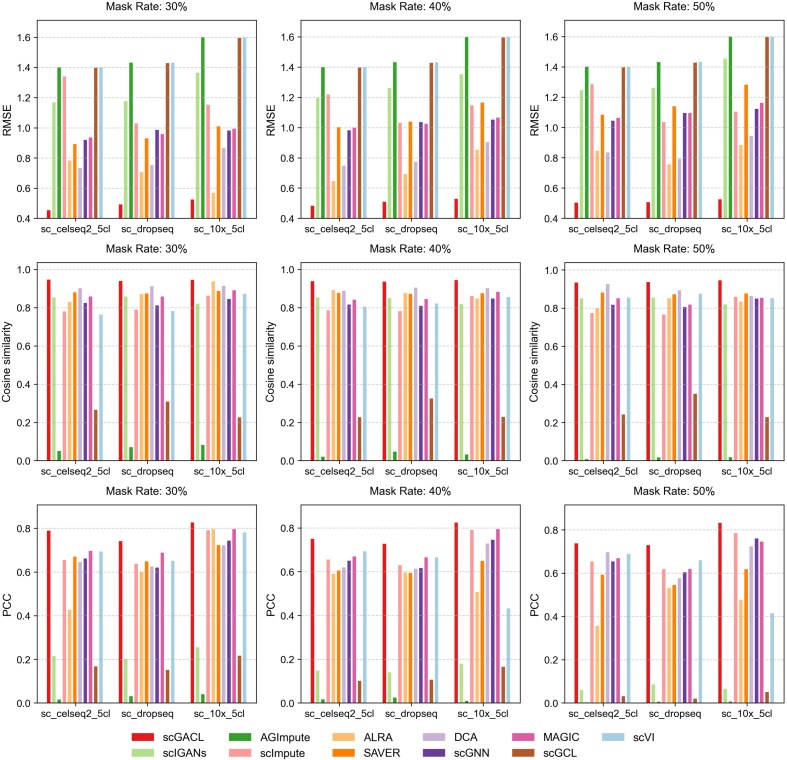
The RMSE, cosine similarity, and PCC between the imputed results and the ground truth.

Having demonstrated the superior performance of scGACL on simulated datasets, we next evaluate whether this advantage translates to real-world scenarios. We test all methods on three real-world scRNA-seq datasets from CellBench [34] (sc_celseq2_5cl, sc_10x_5cl, and sc_dropseq). For each dataset, we independently simulate dropout events by randomly masking 30%, 40%, and 50% of its nonzero expression values. The final zero rates for each dataset under these masking conditions are reported in Supplementary Table S2. After imputing these masked datasets with scGACL and other methods, we calculate the RMSE, cosine similarity, and PCC between the imputed and ground truth values at the masked positions. The results for mask rates of 30%, 40%, and 50% are presented in Fig. 4. Across all datasets and mask rates, scGACL consistently outperforms other methods, achieving the lowest RMSE, highest cosine similarity, and highest PCC. This indicates its superior ability to recover gene expression data in real-world datasets. In contrast, several methods demonstrate notable limitations. AGImpute ranks lowest among all methods, achieving the highest RMSE while its PCC and cosine similarity are both close to zero. Similarly, scGCL exhibits suboptimal performance, with a high RMSE and the second-lowest correlation metrics. Furthermore, under the 50% mask rate, the imputation results of scIGANs exhibit a weak correlation with the ground truth, as indicated by a PCC value approaching zero.

Taken together, these results on both simulated and real-world datasets consistently demonstrate scGACL’s superior performance, confirming its robustness in recovering gene expression data. This superior performance stems from several key factors. First, scGACL employs Gamma-Normal mixture models to identify dropout events for imputation while preserving biological zeros, which directly contributes to minimal RMSE and high correlation metrics. Second, the integration of multi-scale contrastive learning within the GAN framework prevents over-smoothing by preserving cellular heterogeneity while ensuring the imputed data distribution accurately matches the real data. In contrast, the performance limitations of several competing methods can be attributed to specific architectural and methodological choices. scGCL exhibits suboptimal performance due to over-smoothing in its GNNs, which homogenizes cellular representations and leads to imputation that deviates substantially from the ground truth. Similarly, the CNN-based framework of scIGANs is less suited for scRNA-seq data, as it assumes spatial correlations among genes that may not be present. For AGImpute, it utilizes a combination of ZIP, ZINB, and GMMs to detect dropout events; however, many dropout events remain undetected, leading to insufficient imputation. In summary, scGACL’s superiority stems from its integrated design, which simultaneously resolves the core challenges of dropout identification and heterogeneity preservation which competing methods fail to address.

### Cell clustering analysis

**Figure 5:**
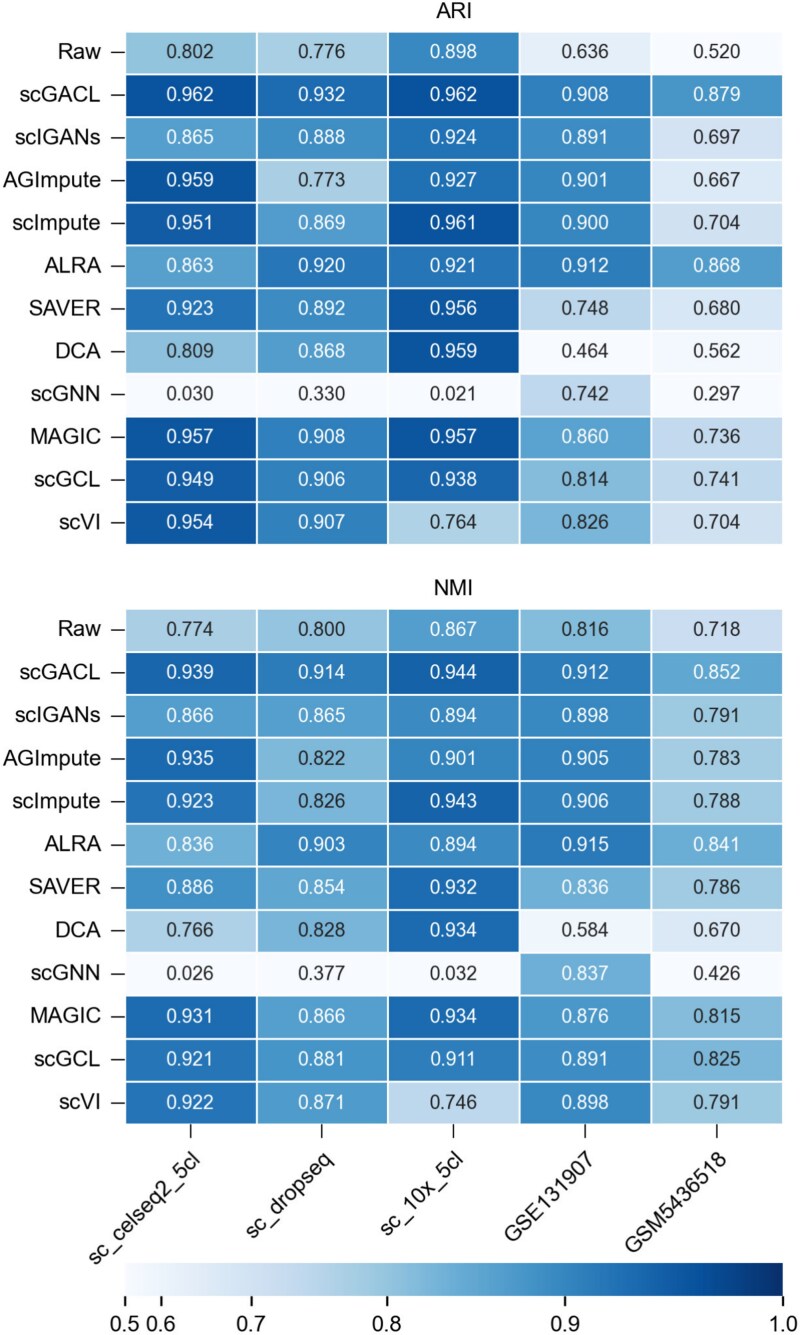
ARI and NMI of raw data and data imputed by scGACL and other baseline methods across five datasets.

Cell clustering is a key part of downstream analysis and is crucial for identifying cell types. Accordingly, we evaluate clustering performance on five datasets with known cell type labels, comprising four human lung adenocarcinoma datasets (sc_celseq2_5cl, sc_10x_5cl, sc_dropseq, and GSE131907 [35]) and one human lipoma dataset (GSM5436518 [36]). We apply the K-means algorithm (with k set to the number of cell types) to cluster the raw and imputed data, and assess the results using ARI and NMI. A key consideration is that scGACL requires cell type information for both dropout identification and model training. To prevent label leakage in this analysis, we perform an initial K-means clustering on the preprocessed data and use the resulting “pre-clustering” labels rather than true cell type labels to guide the imputation task. As shown in Fig. 5, scGACL’s imputation improves clustering performance over the raw data across all five datasets. Specifically, scGACL achieves the highest ARI and NMI on four datasets, only ranking second to ALRA on GSE131907. Moreover, while most imputation methods improve clustering performance (as reflected in higher ARI and NMI compared to raw data), scGNN yields lower ARI and NMI on most datasets. DCA also demonstrates suboptimal performance, as its imputation results on the GSM5436518 dataset yield poorer clustering performance than the raw data. Overall, the robust clustering improvement by scGACL across diverse datasets underscores its capability to recover critical biological distinctions between cell types.

**Figure 6:**
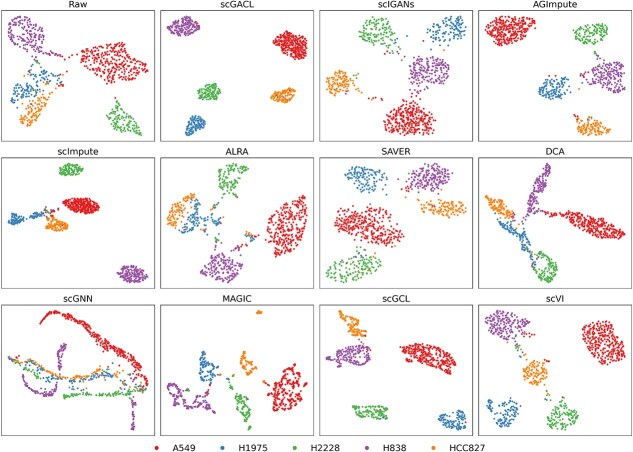
UMAP visualizations of raw data and data imputed by scGACL and other baseline methods on the sc_celseq2_5cl dataset.

Next, we visualize raw and imputed data using Uniform Manifold Approximation and Projection (UMAP) [37] and present the results for five datasets (sc_celseq2_5cl, sc_dropseq, sc_10x_5cl, GSE131907, and GSM5436518) in Fig. 6 and Supplementary Figs S2–S5. The visualization results demonstrate that scGACL’s imputation produces clearer boundaries between distinct cell types compared to raw data. For example, in the sc_celseq2_5cl dataset, the raw data shows poor separation with H1975 and HCC827 cells severely mixed. After scGACL imputation, all five cell types form distinct clusters. However, the UMAP visualizations of scImpute and ALRA still fail to separate the H1975 and HCC827 cell types, which remain partially overlapping. Furthermore, scGNN also shows suboptimal performance, where cells from the five types are mixed together and do not form meaningful clusters. Overall, these observations indicate that scGACL’s imputation leads to clearer visual representations compared to other imputation methods.

### Gene differential expression analysis

High dropout rates in scRNA-seq data severely hinder the identification of differentially expressed genes (DEGs). Data imputation addresses this issue by reducing dropouts to reveal true gene expression patterns. Following scGGAN [16], we apply limma [38] to find DEGs on raw and imputed scRNA-seq data. The criterion for DEGs is that the log fold changes $\ge 1$ or $\le -1$ with adjusted *P*-value $\le 0.05$. Since bulk RNA-seq data is largely unaffected by dropout events, its differential expression analysis results serve as the gold standard. Following Hou *et al.* [4], we use the sc_10x dataset from CellBench for DEGs analysis, and the GSE86337 [39] dataset serves as the matching bulk RNA-seq dataset. The sc_10x dataset contains three lung adenocarcinoma cell lines (H1975, H2228, and HCC827). Therefore, DEGs are identified from three pairwise comparisons: H2228 versus H1975, HCC827 versus H1975, and HCC827 versus H2228.

**Figure 7:**
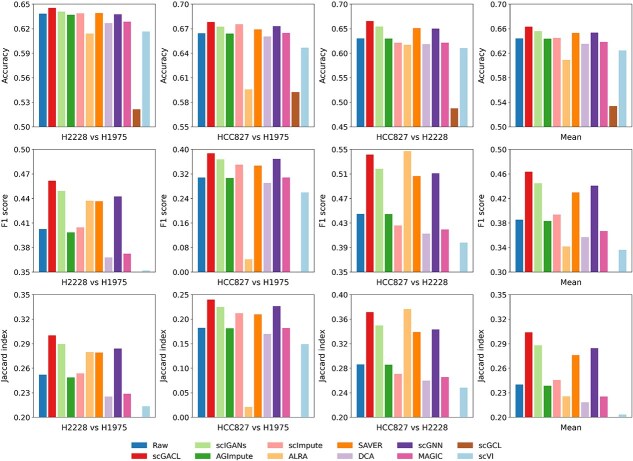
Performance of different methods for DEGs identification across three pairwise comparisons, where the “Mean” column shows the average performance across the three comparisons.

The agreement between DEGs identified from imputed data and the gold standard is quantified using the F1 score, Jaccard index, and accuracy, as shown in Fig. 7. Figure 7 reveals that scGACL achieves the highest scores across all three metrics in two of the three comparisons (H2228 versus H1975 and HCC827 versus H1975). In the third comparison (HCC827 versus H2228), its F1 score (0.542) and Jaccard index (0.371) are slightly lower than those of ALRA (F1 score of 0.547 and Jaccard index of 0.376), though it still attains the highest accuracy. In contrast, ALRA’s performance is unstable, with its metrics falling below the raw data in one comparison (HCC827 versus H1975). Notably, no DEGs are identified from the imputed data of scGCL, resulting in zero F1 and Jaccard scores. This failure stems from its imputation process producing overly homogenized gene expression profiles, which eliminates the variation needed for differential analysis. DCA and scVI perform similarly poorly and worse than raw data, as over-smoothing in their imputation outputs causes most genes to have nearly indistinguishable expression levels, leading to very few detectable DEGs. In summary, these results demonstrate that scGACL effectively overcomes the over-smoothing issue common to other imputation methods, thereby preserving critical biological variance and achieving superior performance in DEGs identification.

### Cell trajectory inference

Inferring cell trajectory from scRNA-seq data allows for the exploration of cell cycle dynamics. Dropout events affect the trajectory inference of scRNA-seq data, and imputation methods can effectively alleviate this phenomenon. To evaluate the performance of imputation methods, we apply these methods to the published Deng dataset [40], a time-course scRNA-seq dataset comprising single cells from ten stages of early mouse development (zygote, early 2-cell, mid 2-cell, late 2-cell, 4-cell, 8-cell, 16-cell, early blastocyst, mid blastocyst, and late blastocyst). Subsequently, we use Monocle2 [41] to visualize trajectories and infer pseudo-time. POS and KOR are used to measure the correlation between the real time labels and the pseudo-time labels.

**Figure 8:**
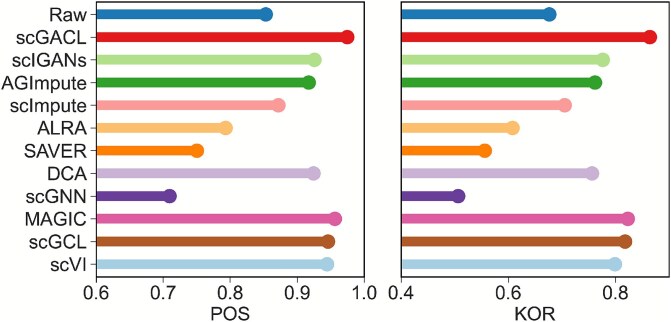
POS (left) and KOR (right) of different imputation methods on the Deng dataset.

**Figure 9:**
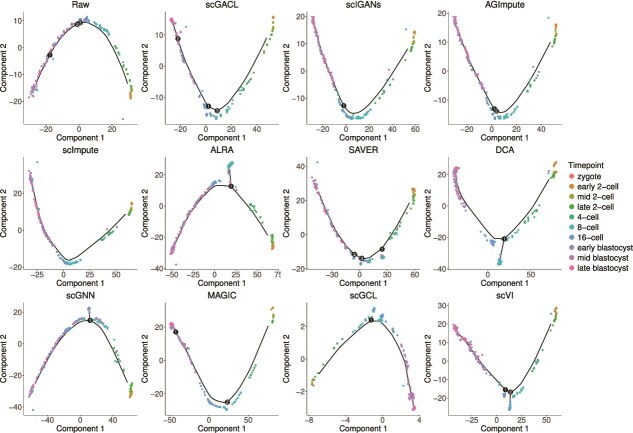
The inferred trajectory from the raw and imputed data, with cell stages indicated in the legend.

Figure 8 presents the POS and KOR of different imputation methods, demonstrating that scGACL achieves the best performance in the cell trajectory inference task. Its POS (0.975) and KOR (0.864) not only surpass those of other imputation methods, but also represent a significant improvement compared to the raw data (POS = 0.853, KOR = 0.676). In contrast, scGNN shows the poorest performance, with its POS and KOR being the lowest among all imputation methods and even lower than those of the raw data. Similarly, ALRA and SAVER exhibit lower POS and KOR values than the raw data. As a result, their imputation adversely affects trajectory inference. In Fig. 9, we visualize the cell trajectories reconstructed by Monocle2 from the raw and imputed data, showing that imputation by scGACL enables more accurate and clearer trajectory inference. Specifically, the trajectory inferred from raw data shows high overlap between cells from the 16-cell and early blastocyst stages, whereas the trajectory inferred from the scGACL-imputed data clearly distinguishes between these two developmental stages. In contrast, the trajectory for scGNN-imputed data shows poor separation, with significant overlap among cells from different time points (e.g. early, mid, and late 2-cell stages). Trajectories based on scImpute, scVI, and MAGIC imputation appear discontinuous and lack smoothness. For SAVER, scIGANs, and AGImpute, the inferred trajectories show partial overlap between cells from the 8-cell and 16-cell stages. Similarly, for ALRA, DCA, and scGCL, the trajectories exhibit mixing between early and mid blastocyst cells. In summary, scGACL’s imputation robustly enhances trajectory inference, yielding superior accuracy (as measured by POS and KOR) and clearer visualization of developmental trajectories.

### Computing resource analysis

To benchmark computing resources, we generate six data subsets from the GSE131907 dataset by first selecting the top 5000 highly variable genes and then downsampling to 500, 1000, 5000, 10 000, 20 000, and 30 000 cells. The runtime and memory usage of each method are then evaluated on these subsets using a 20-core machine with 128GB of RAM.

As shown in Supplementary Fig. S6, several models exhibit poor scalability. AGImpute and scImpute require over a week to process the 10 000-cell and 30 000-cell datasets, respectively, while scGNN fails with an out-of-memory error on the 10 000-cell dataset. Consequently, AGImpute and scGNN are excluded from benchmarks at cell counts $\ge $ 10 000, and scImpute at cell counts $\ge $ 30 000. While DCA, ALRA, and MAGIC are the fastest overall, scGACL is significantly more efficient than other deep learning-based methods like scIGANs, scVI, AGImpute, and scGCL. Furthermore, its memory usage is lower than that of scIGANs, SAVER, and scGNN. Crucially, scGACL’s resource usage grows slowly with increasing cell numbers, demonstrating its excellent scalability for large datasets.

### Ablation study

To assess the contribution of each scGACL component, we perform an ablation study using the VaDE generator as the base architecture. Four variants are created by removing: (i) the adversarial discrimination module; (ii) the cell-level contrastive learning module; (iii) the cell-type-level contrastive learning module; or (iv) the entire multi-task discriminator. We evaluate these variants and the full model on four simulated datasets (Simulated 1–4) and three real-world scRNA-seq datasets (sc_celseq2_5cl, sc_dropseq, and sc_10x_5cl), following the same procedure as in the gene expression data recovery experiment. We evaluate imputation performance using RMSE, PCC, and cosine similarity. For real-world datasets, we randomly mask 50% of nonzero values and assess imputation at the masked positions. As shown in Supplementary Figs S7 and S8, the full scGACL performs best across all metrics and datasets. Removing the entire multi-task discriminator severely degrades performance, and ablating any individual module also leads to a clear drop, confirming each component’s necessity.

To justify the choice of VaDE as the generator, we further compare scGACL with a variant, scGACL-VAE, in which the VaDE module is replaced by a standard VAE while keeping all other components unchanged. We evaluate both models using the same imputation protocol and datasets as above. As summarized in Supplementary Figs S9 and S10, scGACL attains similar RMSE to scGACL-VAE on simulated datasets with lower zero rates (71% and 78%), but clearly lower RMSE and higher PCC and cosine similarity on datasets with higher zero rates (84% and 91%) and on all three real-world datasets. These results indicate that using VaDE as the generator rather than a standard VAE leads to more accurate imputation.

## Discussion

In this work, we propose scGACL, a novel framework that combines a GAN with multi-scale contrastive learning. The GAN architecture is designed to ensure that the distribution of imputed data closely matches that of the real data. Meanwhile, cell-level contrastive learning preserves fine-grained cell-to-cell heterogeneity, and cell-type-level contrastive learning maintains macroscopic biological variation across different cell types. Together, these mechanisms improve imputation accuracy and overcome the over-smoothing problem.

Experimental results across diverse simulated and real-world scRNA-seq datasets demonstrate the excellent performance of scGACL. It achieves the highest accuracy and F1 score with a low FPR in dropout identification, effectively preventing over-imputation. Subsequently, it yields the most accurate gene expression recovery, as evidenced by the lowest RMSE, highest PCC, and highest cosine similarity across all datasets. This high-quality output robustly enhances downstream analyses: scGACL attains the best ARI and NMI in cell clustering across four real-world datasets, enables more accurate identification of DEGs, and achieves the highest POS and KOR scores for trajectory inference. In summary, scGACL’s imputation effectively recovers gene expression and thereby significantly enhances downstream analyses. Future work could extend scGACL to integrate multi-omics data, such as single-cell ATAC-seq and single-cell methylation profiles. Leveraging this additional biological prior knowledge would further enhance imputation performance.

Key Points

- scGACL employs a Gamma-Normal mixture distribution to accurately identify dropout events. Validation on 8 simulated datasets demonstrates that scGACL achieves high dropout detection accuracy with low false positive rates.
- scGACL integrates a generative adversarial network with multi-scale contrastive learning, where adversarial training ensures the imputed data distribution approximates the real data distribution, while multi-scale contrastive learning preserves both fine-grained cell-to-cell heterogeneity and macroscopic biological variations across cell types.
- scGACL accurately recovers gene expression profiles in both simulated and real-world datasets, achieving minimal error and maximal correlation between imputed and ground truth values, outperforming state-of-the-art methods.
- scGACL improves downstream analyses, including cell clustering, differential expression analysis, and trajectory inference.

## Supplementary Material

bbag018_Supplementary_File

## Data Availability

scGACL is available at: https://github.com/Hhjyl/scGACL. The CellBench datasets are available at: https://www.ncbi.nlm.nih.gov/geo/query/acc.cgi?acc=GSE118767. The Deng dataset can be accessed at https://figshare.com/articles/software/scRNMF/23725986?file=41653401. The GSE131907 dataset is available at https://www.ncbi.nlm.nih.gov/geo/query/acc.cgi?acc=GSE131907. The GSM5436518 dataset is obtained from https://db.cngb.org/cdcp/dataset/SCDS0000567/.
