# Supplementary material for: scGACL: a generative adversarial network with multi-scale contrastive learning for accurate single-cell RNA sequencing imputation
Source: Brief Bioinform. 2026 Feb 3;27(1):bbag018. doi: 10.1093/bib/bbag018 (PMC12866930; doi:10.1093/bib/bbag018)
Supplement: bbag018_Supplementary_File [file bbag018_supplementary_file.pdf]

Supplementary Materials -

scGACL: a generative adversarial network with multi-scale contrastive learning for accurate scRNA-seq imputation

1. Supplementary Tables

Dataset	Number of Cells	Number of Genes	Number of Cell Types	Zero Rate	Evaluation	Source
Simulated 1	500	1000	4	91%	Evaluation of dropout identification; Gene expression data recovery; Ablation study	Splatter [1]
Simulated 2	500	1000	4	84%	Evaluation of dropout identification; Gene expression data recovery; Ablation study	Splatter [1]
Simulated 3	500	1000	4	78%	Evaluation of dropout identification; Gene expression data recovery; Ablation study	Splatter [1]
Simulated 4	500	1000	4	71%	Evaluation of dropout identification; Gene expression data recovery; Ablation study	Splatter [1]
Simulated 5	500	1000	4	63%	Evaluation of dropout identification; Gene expression data recovery	Splatter [1]

Simulated 6	500	1000	4	55%	Evaluation of dropout identification; Gene expression data recovery	Splatter [1]
Simulated 7	500	1000	4	48%	Evaluation of dropout identification; Gene expression data recovery	Splatter [1]
Simulated 8	500	1000	4	42%	Evaluation of dropout identification; Gene expression data recovery	Splatter [1]
sc_celseq2_5cl	909	12653	5	62%	Gene expression data recovery; Cell clustering analysis; Ablation study	CellBench [2]
sc_dropseq	225	15127	3	67%	Gene expression data recovery; Cell clustering analysis; Ablation study	CellBench [2]
sc_10x_5cl	3918	11786	5	67%	Gene expression data recovery; Cell clustering analysis; Ablation study	CellBench [2]
GSE131907	208506	29634	9	96%	Cell clustering analysis; Computing resource analysis	GSE131907
GSM5436518	7362	26202	7	94%	Cell clustering analysis	GSM5436518
sc_10x	902	11257	3	54%	Gene differential expression analysis	CellBench [2]
Deng	286	18884	10	56%	Cell trajectory inference	[3]

Supplementary Table S1: scRNA-seq datasets used in the experiments. Due to the large size of the GSE131907 dataset, we randomly select 5,000 cells for cell

clustering analysis. The cell type labels for the GSM5436518 dataset are obtained from the Cell-omics Data Coordinate Platform [4].

Dataset	Original zero rate	Zero rate after masking 30% of non-zero values	Zero rate after masking 40% of non-zero values	Zero rate after masking 50% of non-zero values
sc_celseq2_5cl	62%	74%	77%	81%
sc_dropseq	67%	77%	80%	83%
sc_10x_5cl	67%	77%	80%	83%

Supplementary Table S2: Summary of the final zero rates for the three real-world scRNA-seq datasets after simulating dropout events. For each dataset, 30%, 40%, and 50% of the nonzero values are randomly masked to generate three levels of sparsity.

2. Supplementary Figures

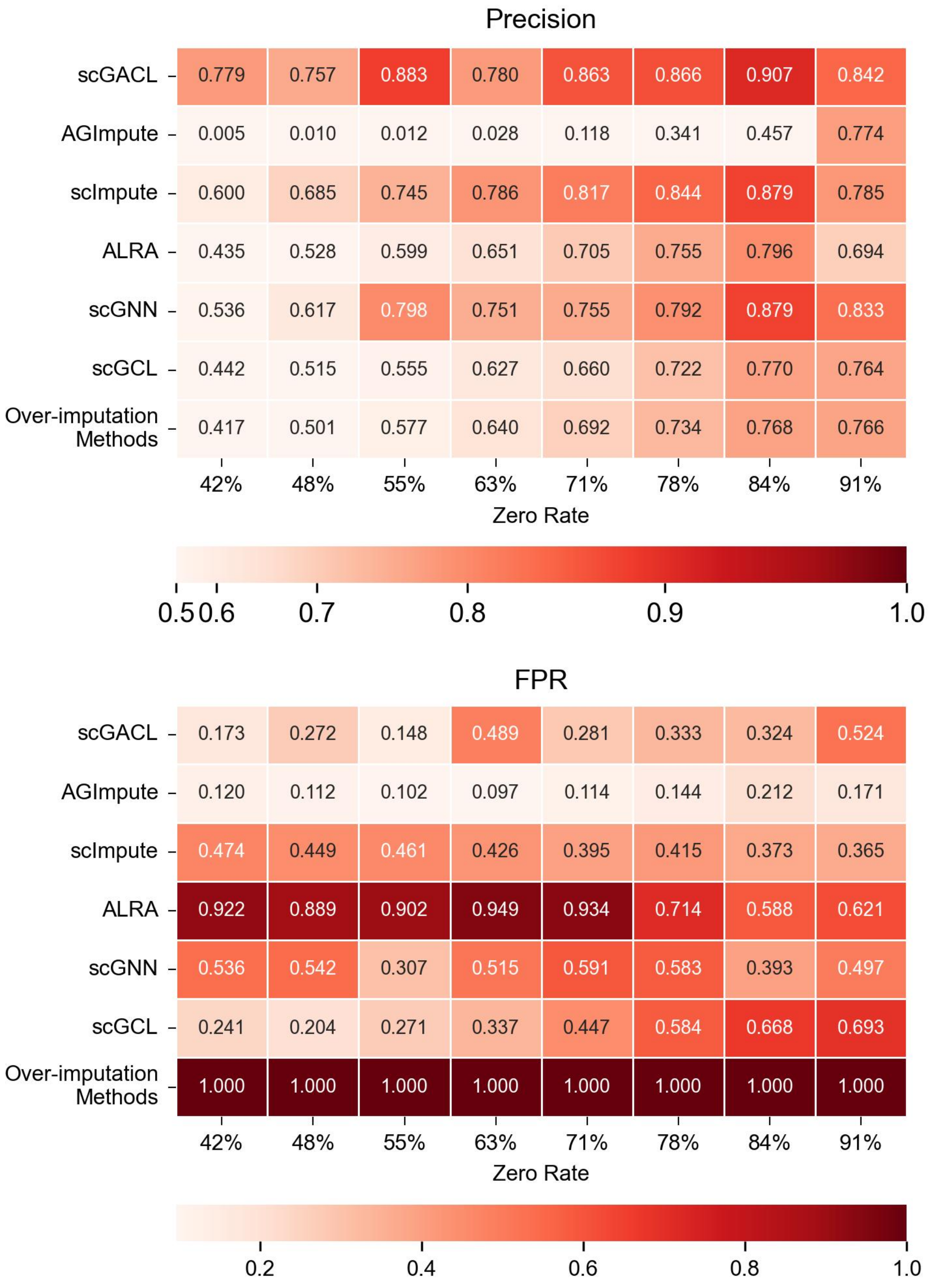

Supplementary Figure S1: Comparison of dropout identification performance on eight simulated datasets. Precision and false positive rate (FPR) are shown as heatmaps, with rows representing imputation methods and columns indicating zero rates of simulated datasets. The "Over-imputation Methods" represent a group of five methods (scIGANs, SAVER, DCA, scVI, and MAGIC) that adopt a uniform strategy of imputing all zero values without distinguishing dropout events from biological zeros, consequently producing identical performance metrics.

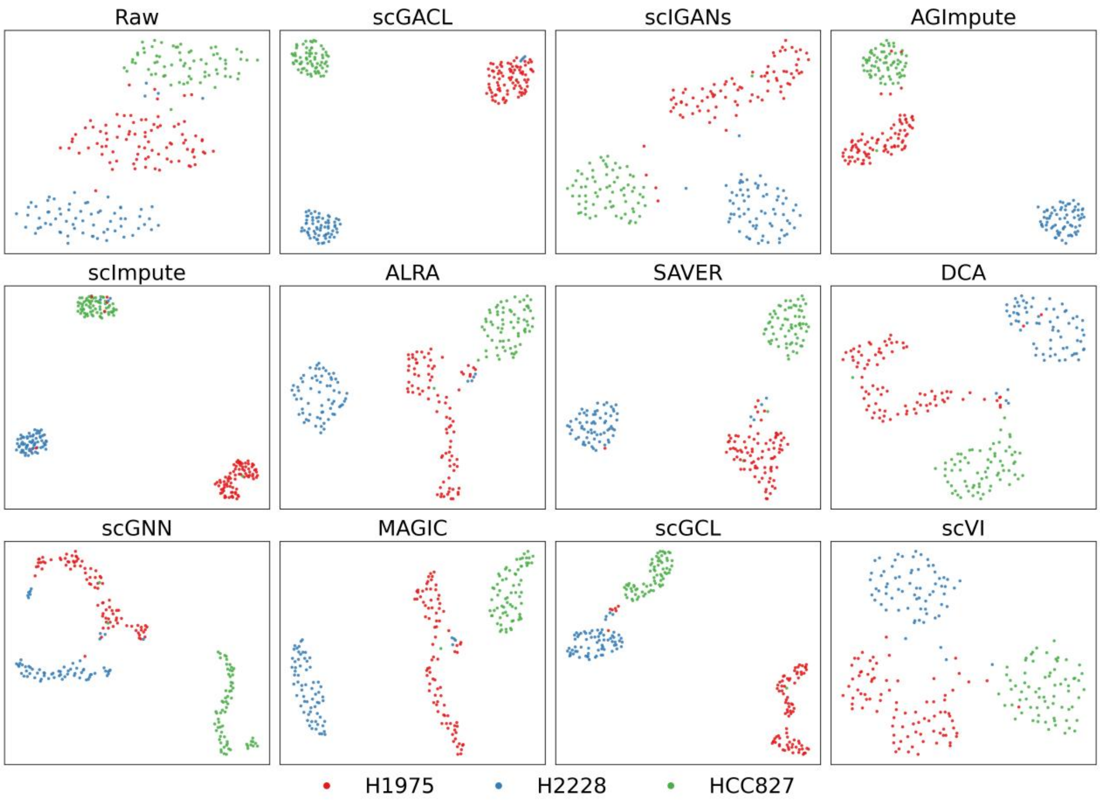

Supplementary Figure S2: UMAP visualizations of raw data and imputed data obtained from scGACL and baseline methods on the sc_dropseq dataset.

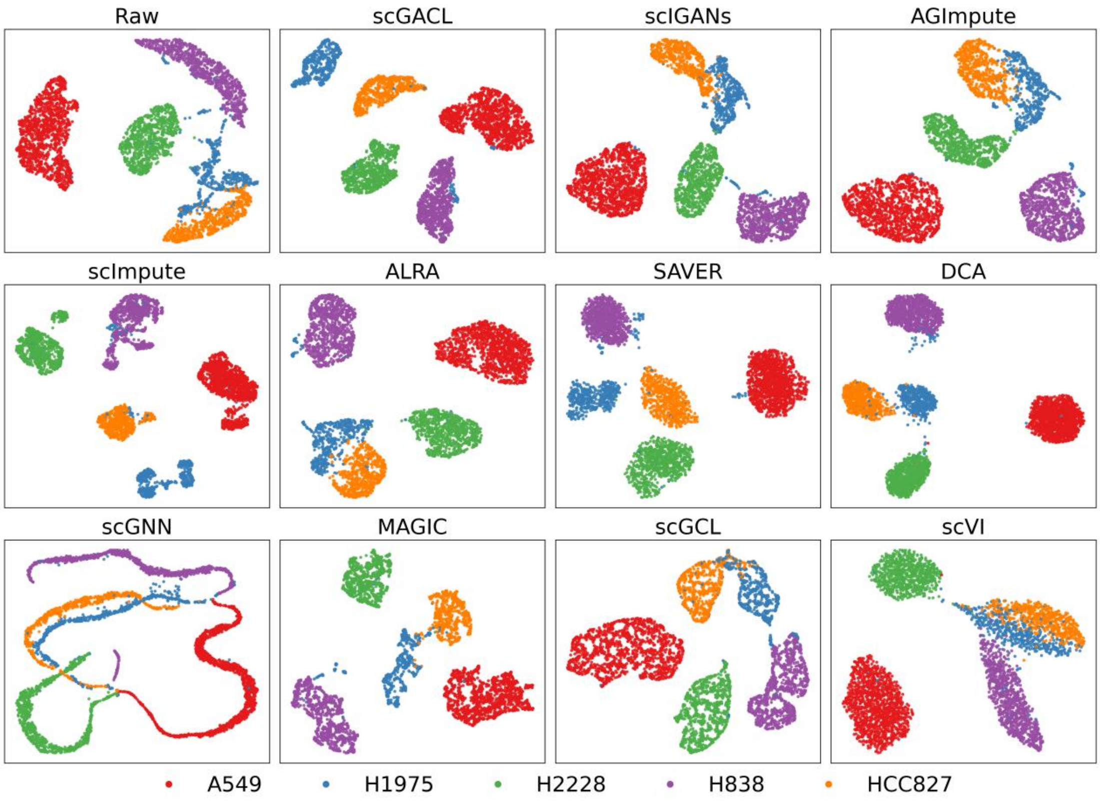

Supplementary Figure S3: UMAP visualizations of raw data and imputed data obtained from scGACL and baseline methods on the sc_10x_5cl dataset.

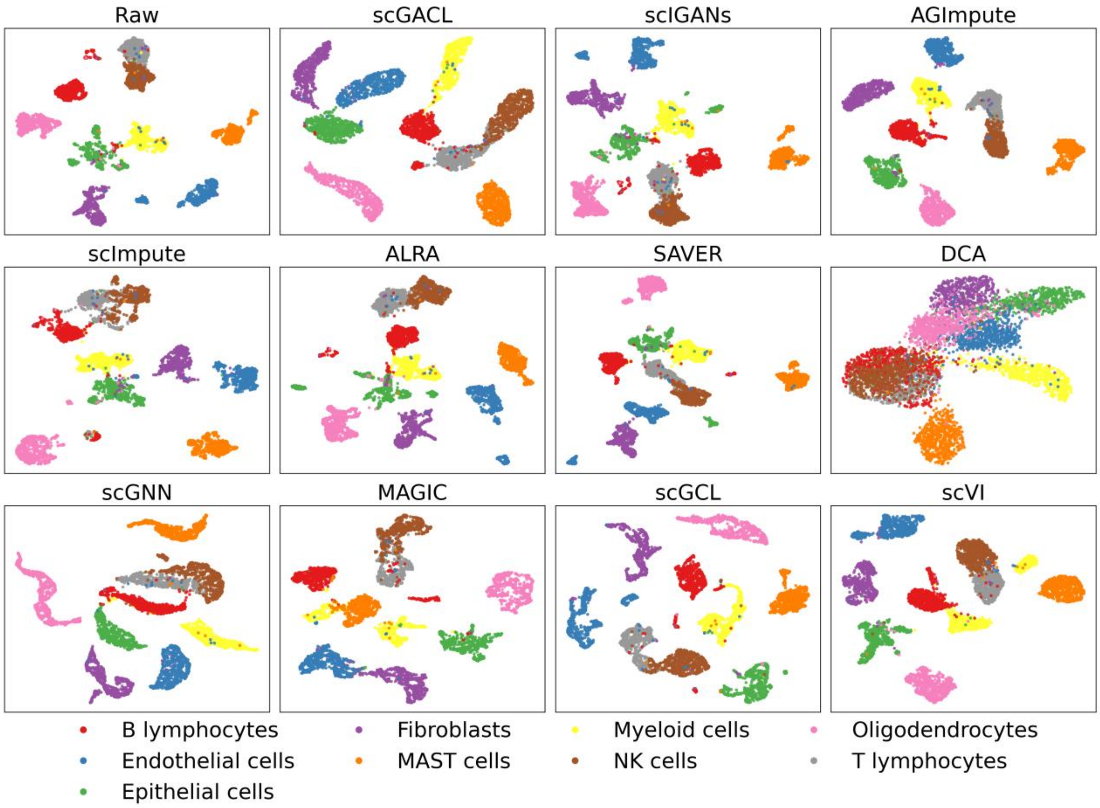

Supplementary Figure S4: UMAP visualizations of raw data and imputed data obtained from scGACL and baseline methods on the GSE131907 dataset.

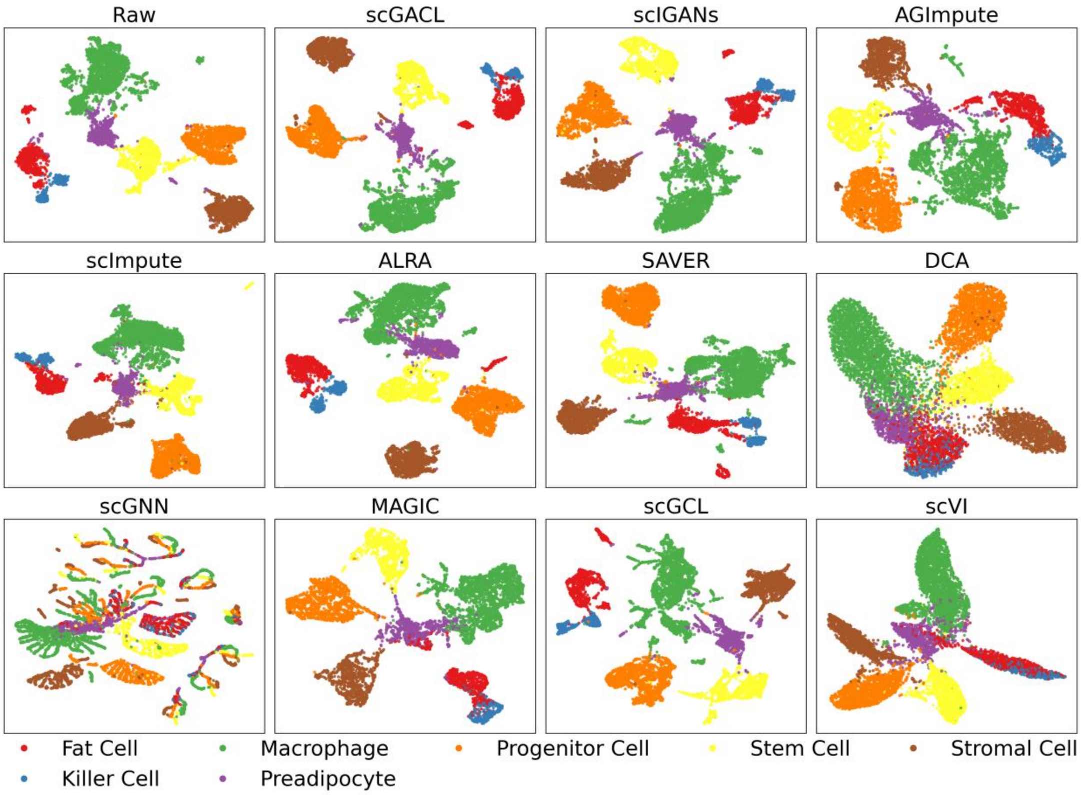

Supplementary Figure S5: UMAP visualizations of raw data and imputed data obtained from scGACL and baseline methods on the GSM5436518 dataset.

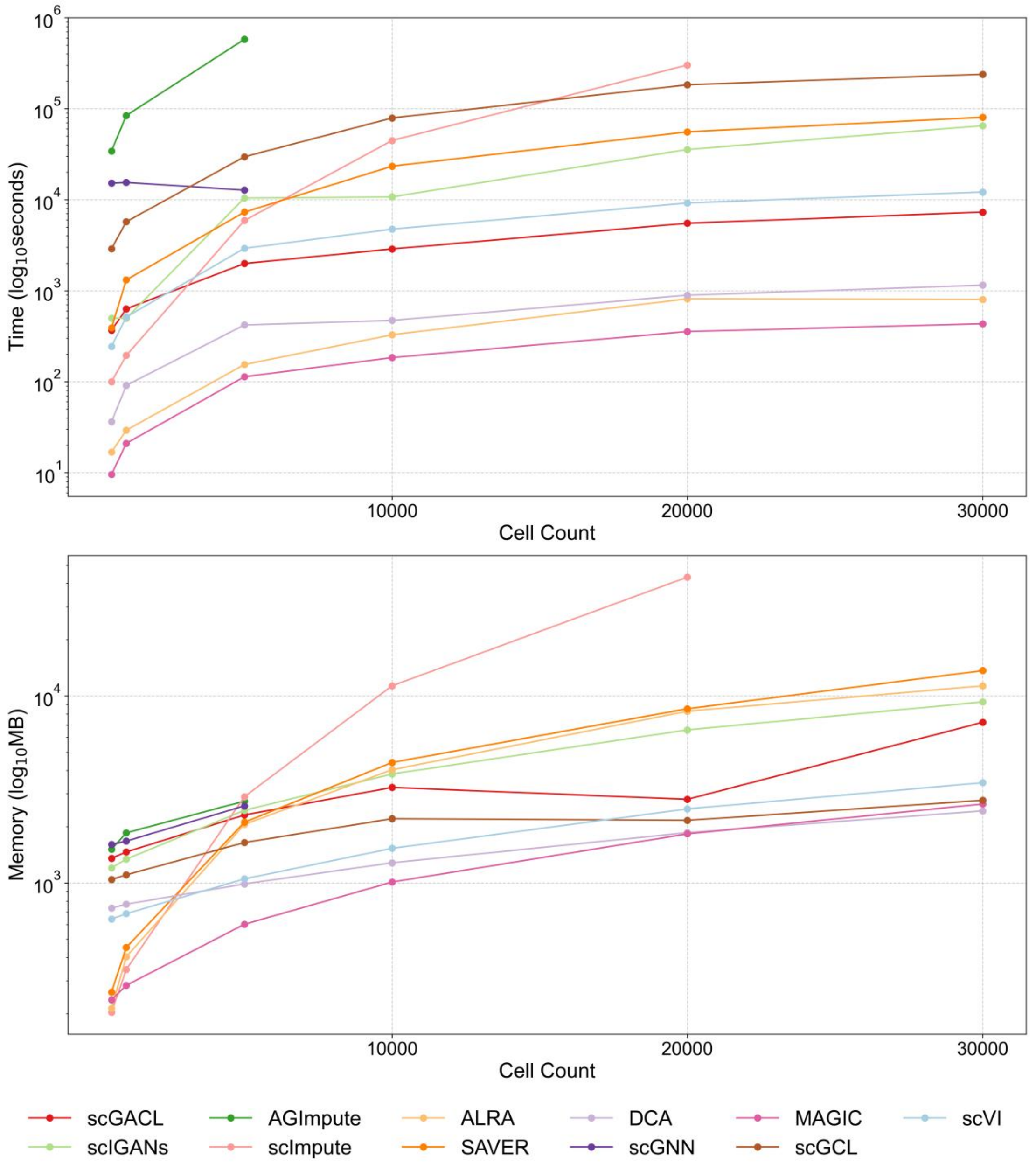

Supplementary Figure S6: Runtime and memory usage of all imputation methods on datasets with different cell counts.

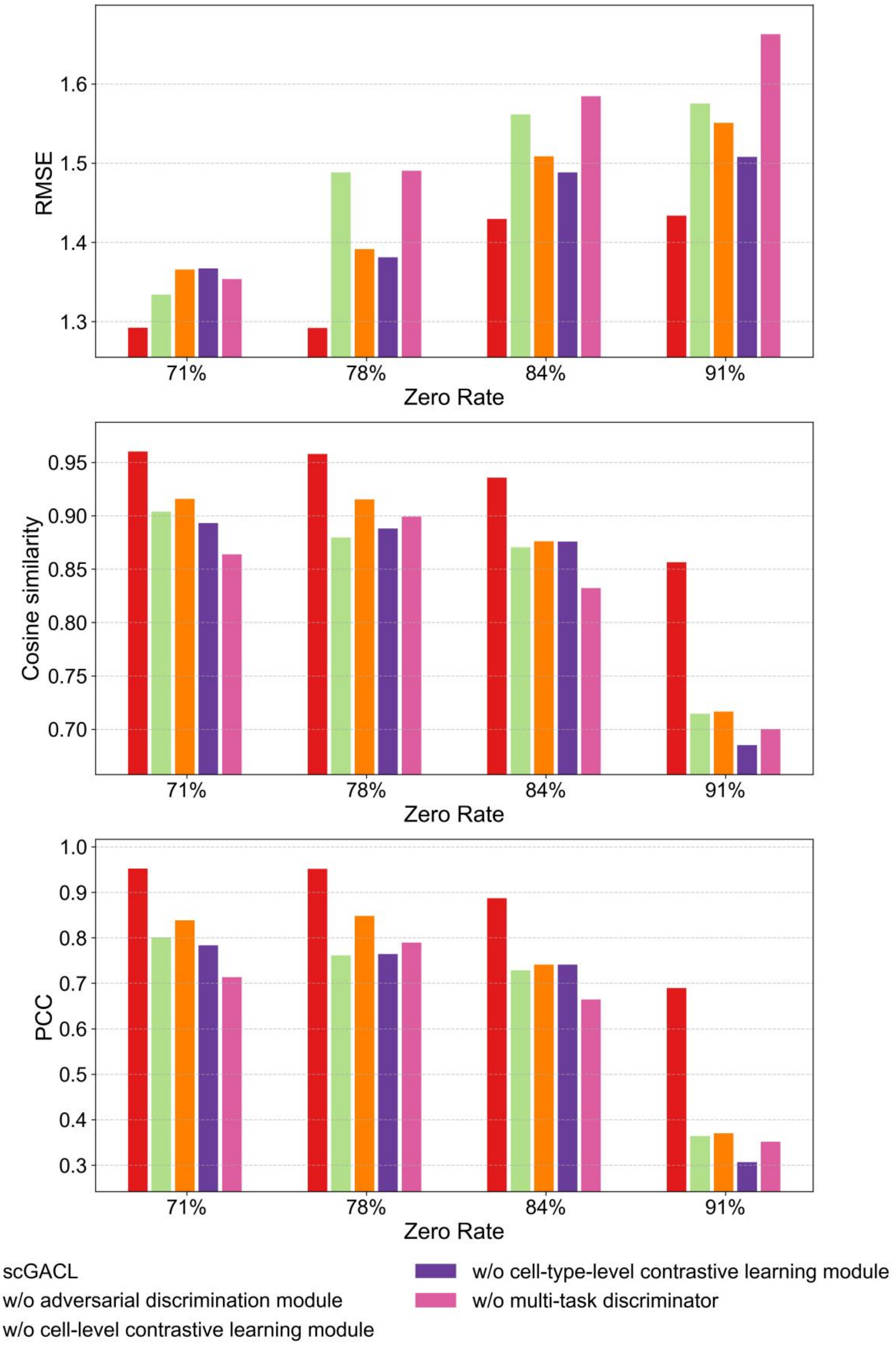

Supplementary Figure S7: Results of the ablation study on the simulated datasets. The x-axis represents the zero rate (71%, 78%, 84%, and 91%) of the simulated datasets.

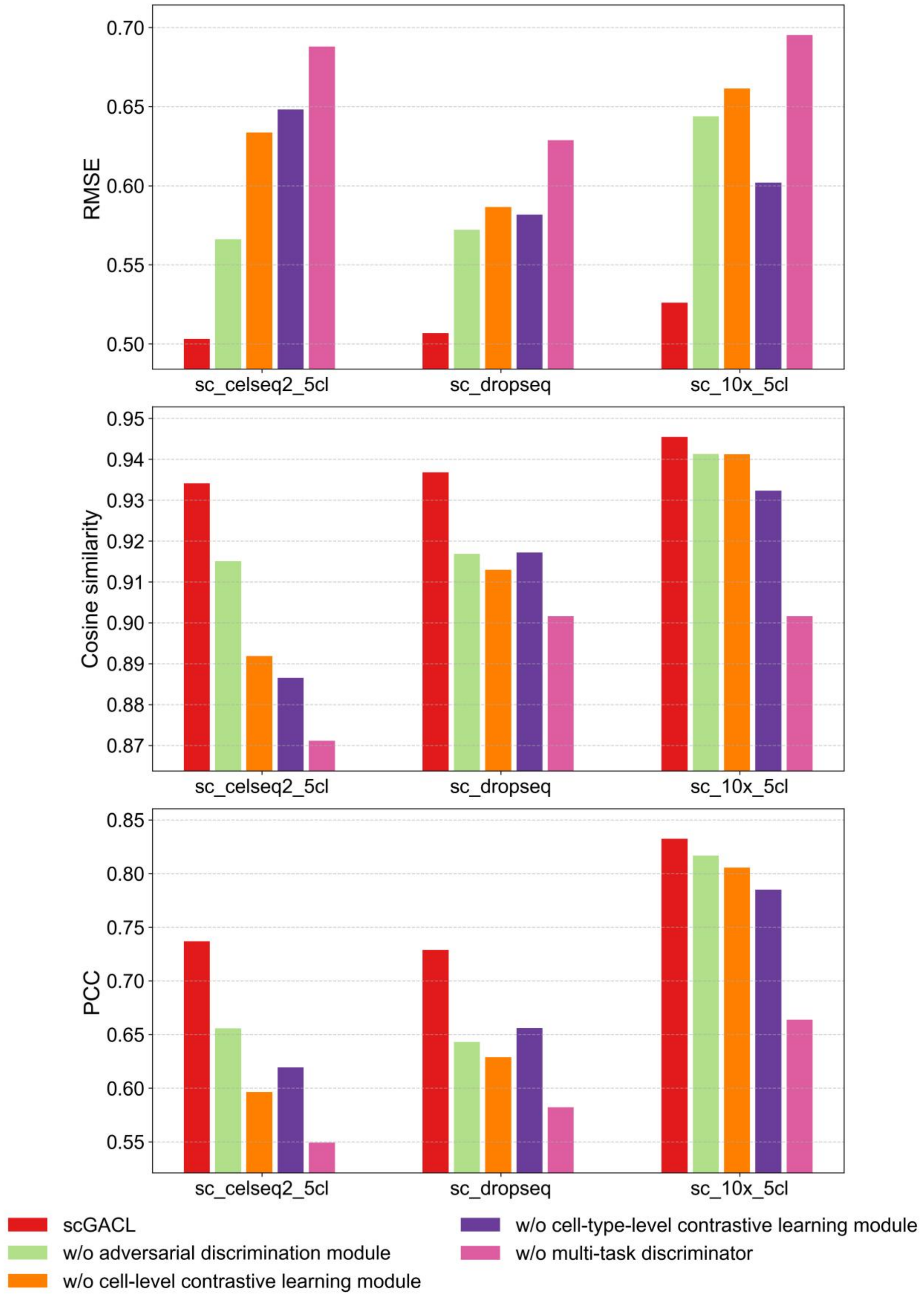

Supplementary Figure S8: Comparison of scGACL and its ablation variants under 50% masking on three real-world datasets. In the sc_celseq2_5cl, sc_dropseq, and sc_10x_5cl datasets, 50% of the nonzero values are randomly masked and then imputed by scGACL and its module-removed variants. Imputation performance is evaluated at the masked positions using RMSE, PCC, and cosine similarity between imputed and ground-truth values.

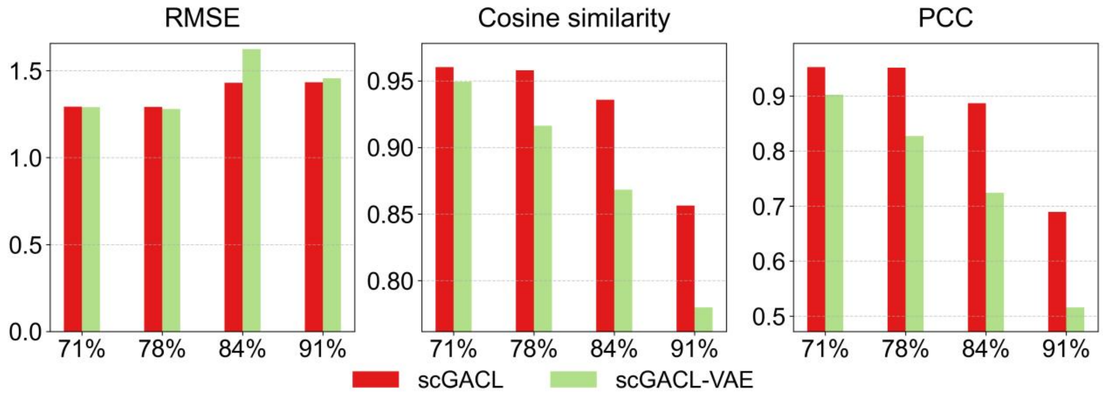

Supplementary Figure S9: Comparison of scGACL with VaDE and VAE generators on simulated datasets. To evaluate the impact of the generator choice, we compare the original scGACL model (with a VaDE generator) against a variant using a standard VAE generator (scGACL-VAE) on four simulated scRNA-seq datasets. The x-axis represents the zero rate (71%, 78%, 84%, and 91%) of the simulated datasets.

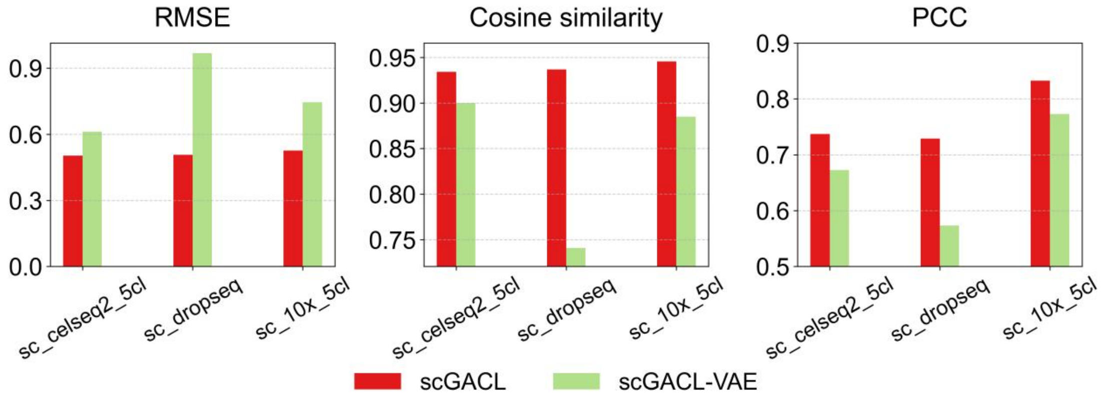

Supplementary Figure S10: Comparison of scGACL with VaDE and VAE generators on three real-world datasets. To evaluate the impact of the generator choice, we compare the original scGACL model (with a VaDE generator) against a variant using a standard VAE generator (scGACL-VAE) on three real-world scRNA-seq datasets. In the sc_celseq2_5cl, sc_dropseq, and sc_10x_5cl datasets, 50% of the nonzero values are randomly masked and then imputed by scGACL and scGACL-VAE. Imputation performance is evaluated at the masked positions using RMSE, PCC, and cosine similarity between imputed and ground-truth values.

3.Supplementary Note

Supplementary Note S1: Data preprocessing

The preprocessing pipeline involves filtering for low-quality cells and genes, followed by normalization and log-transformation. Finally, for datasets with more than 5,000 genes, we use Scanpy [5] to select the top 5,000 highly variable genes (HVGs) for imputation.

Supplementary Note S2: Objective function of Variational Deep Embedding (VaDE)

The VaDE model is defined by two core processes: a generative process and an inference process [6]. These two processes are jointly optimized by maximizing the evidence lower bound (ELBO).

The generative process defines a probabilistic model for generating a sample x. We assume that there are K clusters. A cluster indicator k is sampled from a categorical distribution:

$$p(k) = \mathrm{Cat}(k|\pi) \tag{1}$$

$$\pi = (\pi_k)_{k=1}^{K} \tag{2}$$

$$\sum_{k=1}^{K} \pi_k = 1 \tag{3}$$

where π_k denotes the prior probability of cluster k. Conditioned on cluster k, a continuous latent variable z is sampled from a cluster-specific Gaussian distribution:

$$p(z|k) = \mathcal{N}\left(z|\mu_k, \sigma_k^2 I\right) \tag{4}$$

where μ_k and σ_k^2 are the mean and variance parameters for cluster k, **I** is the identity matrix. Finally, a sample x is generated from a probabilistic decoder network:

$$p(x|z) = \mathcal{N}(x|\mu_x, \sigma_x^2 I) \tag{5}$$

$$[\mu_x; \log\sigma_x^2] = h(z; \theta) \tag{6}$$

where $h(z;\theta)$ representing the decoder network parameterized by θ. The joint distribution over all variables is thus:

$$p(x, z, k) = p(x|z)p(z|k)p(k) \tag{7}$$

Therefore, the ELBO of the marginal log-likelihood can be obtained according to the Jensen's inequality as

$$\begin{aligned} \log p(x) &= \log \int_z \sum_k p\,(x, z, k) dz \\ &\geq \langle -\log q(z, k|x) + \log p(x, z, k)\rangle_{q(z,k|x)} \\ &= \mathrm{ELBO}. \end{aligned} \tag{8}$$

The ELBO can be rewritten as:

$$\mathcal{L}_{\mathrm{ELBO}} = \mathrm{E}_{q(z,k|x)}[\log p(x|z)] - \mathrm{D}_{\mathrm{KL}}\big(q(z,k|x)||p(z,k)\big). \tag{9}$$

The inference process aims to approximate the true but intractable posterior distribution $p(z,k|x)$. To this end, we introduce a variational distribution $q(z,k|x)$ parameterized by an encoder network $g(\phi)$. For computational efficiency, we adopt the mean-field assumption, so $q(z,k|x)$ can further be factorized as

$$q(z, k|x) = q(z|x)q(k|x) \tag{10}$$

where $q(z|x)$ follows a Gaussian distribution

$$q(z|x) = \mathcal{N}(z|\tilde{\mu}, \tilde{\sigma}^2 I) \tag{11}$$

with parameters $\tilde{\mu}$ and $\tilde{\sigma}^2$ that are calculated by encoder network g with input x and parameter ϕ:

$$[\tilde{\mu}; \log\tilde{\sigma}^2] = g(x; \phi) \tag{12}$$

To optimize the ELBO by Stochastic Gradient Variational Bayes (SGVB) [7], we need to draw samples from $q(z|x)$. we can adopt the reparameterization trick such that the lth sample from $q(z|x)$ can be acquired through a differentiable transformation as

$$z^{(l)} = \tilde{\mu} + \tilde{\sigma} \odot \epsilon^{(l)} \tag{13}$$

where $\epsilon \sim N(0, I)$ is an auxiliary variable, $\odot$ denotes an element-wise product. Then, the sampled $z^{(l)}$ can be substituted into (6) to obtain $\mu_x^{(l)}$ and $\sigma_x^{2(l)}$.

According to the SGVB and reparameterization trick, the ELBO can be obtained by

$$\begin{aligned} \mathcal{L}_{\mathrm{ELBO}} &= \frac{1}{L}\sum_{l=1}^{L}\sum_{d=1}^{D} x_d \log\left(\mu_{xd}^{(l)} + \sigma_{xd}^{2(l)}\epsilon_d^{(l)}\right) \\ &+ (1 - x_d)\log\left(1 - \left(\mu_{xd}^{(l)} + \sigma_{xd}^{2(l)}\epsilon_d^{(l)}\right)\right) \\ &- \frac{1}{2}\sum_{k=1}^{K} q\,(k|x)\sum_{m=1}^{M}\left(\log\sigma_{km}^2 + \frac{\tilde{\sigma}_m^2}{\sigma_{km}^2} + \frac{(\tilde{\mu}_m - \mu_{km})^2}{\sigma_{km}^2}\right) \\ &+ \sum_{k=1}^{K} q\,(k|x)\log\frac{\pi_k}{q(k|x)} + \frac{1}{2}\sum_{m=1}^{M}(1 + \log\tilde{\sigma}_m^2) \end{aligned} \tag{14}$$

where L indicates the number of Monte Carlo samples; D denotes the dimensionality of x, μ_x and σ_x; M indicates the dimensionality of μ_k, σ_k, $\tilde{\mu}$ and $\tilde{\sigma}$.

Supplementary Note S3: Generation steps and parameters for simulated scRNA-seq data

We used Splatter[1] to generate simulated scRNA-seq data. Following the generation steps described in the scIMC, we first generated a complete dataset (Ground Truth) comprising 500 cells and 1,000 genes without dropout events. Subsequently, we generated datasets with dropout events by applying the following parameter settings: group.prob = c(0.25, 0.25, 0.25, 0.25), mean.shape = 0.3, mean.rate = 0.02, de.prob = 0.5, de.facLoc = 0.5, dropout.shape = 0.5, and dropout.type = 'experiment'. Specifically, we varied the dropout.mid parameter from 1 to 6 in steps of 1 to simulate six datasets with zero expression rates of 0.78, 0.71, 0.63, 0.55, 0.48, and 0.42, respectively. We then generated a dataset with a zero expression rate of 0.84 by modifying dropout.mid to 0.2 and dropout.shape to 0.5, while keeping other parameters unchanged. Finally, we obtained a dataset with a zero expression rate of 0.91 by setting dropout.mid to 0.05 and dropout.shape to 0.85, with all other parameters held constant.

Supplementary Note S4: Evaluation metrics

In this section, the gene expression matrix (ground truth) is denoted as X, and $\hat{X}$ is the imputed matrix. To evaluate the performance of different imputation methods in recovering gene expression data, we use root mean square error (RMSE), Pearson correlation coefficient (PCC) and cosine similarity (CS). Then we evaluate the imputed results from different methods based on their performance in three downstream tasks. For clustering, we use the Adjusted Rand Index (ARI) and Normalized Mutual Information (NMI). For the identification of differentially expressed genes (DEGs), we employ Accuracy (ACC), F1-score, and the Jaccard index. For cell trajectory inference, the performance is measured by the pseudo-temporal ordering score (POS) and Kendall's rank correlation score (KOR) metrics. The above metrics are described in details as follows.

1.RMSE. It measures the difference between the imputed values and the ground truth. RMSE is defined as:

$$\mathrm{RMSE}(X, \hat{X}) = \sqrt{\frac{1}{n}\sum_{i=1}^{n}(\hat{X}_i - X_i)^2} \tag{15}$$

2.PCC. Its purpose is to measure the level of linear correlation between the imputed values and the ground truth. The PCC is defined by:

$$\rho_{X,\hat{X}} = \frac{E(X\hat{X}) - E(X)E(\hat{X})}{\sqrt{E(X^2)E^2(X)}\sqrt{E(\hat{X}^2)E^2(\hat{X})}} \tag{16}$$

where $E(X)$ represents the mean of X.

3.CS. Its purpose is to measure the similarity in direction between the imputed values and the ground truth. The CS is defined by:

$$\mathrm{CS}(X, \hat{X}) = (X \cdot \hat{X}) \,/\, (||X||\ ||\hat{X}||) \tag{17}$$

4.ARI. It assesses the similarity between two clusters (predicted vs. true labels) by measuring the fraction of correctly classified pairs of elements [8]. For the given true label set $L = \{l_1, l_2, l_i ... l_n\}$ and predicted label set $U = \{u_1, u_2, u_j ... u_n\}$, it is calculated as:

$$\mathrm{ARI} = \frac{\sum_{ij}\binom{n_{ij}}{2} - \left[\sum_i \binom{a_i}{2} \sum_j \binom{b_j}{2}\right] / \binom{n}{2}}{\frac{1}{2}\left[\sum_i \binom{a_i}{2} + \sum_j \binom{b_j}{2}\right] - \left[\sum_i \binom{a_i}{2} \sum_j \binom{b_j}{2}\right] / \binom{n}{2}} \tag{18}$$

where n_{ij} is the number of pairs of elements that are in the same cluster in L and in the same cluster in U, a_i is the number of elements in cluster l_i in L, b_j is the number of elements in cluster u_j in U, and the symbol (.) denotes the binomial

coefficient.

5.NMI. It measures the mutual dependence between two clustering results while taking into account the differences in their sizes [9], calculated as:

$$\mathrm{NMI} = \frac{\mathrm{MI(L,U)}}{\sqrt{\mathrm{H(L)H(U)}}} \tag{19}$$

$$MI(L,U) = \sum_{\mathrm{i}} \sum_{j} P\left(l_i \cap u_j\right) log \frac{P(l_i \cap u_j)}{P(l_i)P(u_j)} \tag{20}$$

$$H(L) = -\sum_{\mathrm{i}} P\,(l_i) \mathrm{log} P(l_i) \tag{21}$$

$$H(U) = -\sum_{j} P\left(u_j\right) \mathrm{log} P\left(u_j\right) \tag{22}$$

where $p\left(l_i \cap u_j\right)$ represents the probability that a sample belongs to both cluster l_i in L and cluster u_j in U. $P(l_i)$ and $P\left(u_j\right)$ are the probabilities of cluster l_i and u_j, respectively.

6.ACC. It measures the overall proportion of genes that are correctly classified as either differentially expressed or non-differentially expressed. This is formally defined using the following terms:
True Positives (TP): The number of genes correctly identified as DEGs.
True Negatives (TN): The number of genes correctly identified as non-DEGs.
False Positives (FP): The number of non-DEGs incorrectly identified as DEGs.
False Negatives (FN): The number of DEGs that are missed by the method.
Then ACC is calculated as:

$$\mathrm{ACC} = (\mathrm{TP} + \mathrm{TN}) / (\mathrm{TP} + \mathrm{TN} + \mathrm{FP} + \mathrm{FN}) \tag{23}$$

7.F1 score. The F1 score represents the harmonic mean of Precision and Recall.

$$\mathrm{Precision} = \mathrm{TP} / (\mathrm{TP} + \mathrm{FP}) \tag{24}$$

$$\mathrm{Recall} = \mathrm{TP} / (\mathrm{TP} + \mathrm{FN}) \tag{25}$$

Then F1 score is calculated as:

$$\mathrm{F1\ score} = 2 \cdot \frac{\mathrm{Precision} \cdot \mathrm{Recall}}{\mathrm{Precision} + \mathrm{Recall}} \tag{26}$$

8.Jaccard Index. The Jaccard index measures the similarity between the set of predicted DEGs and the set of true DEGs. It is defined by:

$$\mathrm{Jaccard\ Index} = \frac{|\mathrm{G} \cap \mathrm{O}|}{|\mathrm{G} \cup \mathrm{O}|} \tag{27}$$

where G is the set of genes predicted as DEGs and O is the set of true DEGs.

9.POS. POS can be used to evaluate the performance of reconstructing pseudo-times [10]. The formula of POS is:

$$\mathrm{POS} = \sum_{i=1}^{n-1} \sum_{j>i} g(i, j) \tag{28}$$

where n is the number of samples, g(i, j) is a score that characterizes how well the order of the i-th and j-th cells in the ordered path matches their expected order based on the external information.

10.KOR. It is used to measure the degree of correspondence between two rankings [9]. It is defined as:

$$\tau = \frac{4P}{n(n-1)} - 1 \tag{29}$$

where n is the number of samples, and P is the sum of the number of samples ranked after the given sample by both rankings.

References

[1] Luke Zappia, Belinda Phipson, and Alicia Oshlack. Splatter: simulation of single-cell rna sequencing data. Genome biology, 18(1):174, 2017.

[2] Luyi Tian, Xueyi Dong, Saskia Freytag, Kim-Anh Lˆe Cao, Shian Su, Abolfazl JalalAbadi, Daniela Amann-Zalcenstein, Tom S Weber, Azadeh Seidi, Jafar S Jabbari, et al. Benchmarking single cell rna-sequencing analysis pipelines using mixture control experiments. Nature methods, 16(6):479–487, 2019.

[3] Qiaolin Deng, Daniel Ramsk¨old, Bj¨orn Reinius, and Rickard Sandberg. Single-cell rna-seq reveals dynamic, random monoallelic gene expression in mammalian cells. Science, 343(6167):193–196, 2014.

[4] Yuejiao Li, Tao Yang, Tingting Lai, Lijin You, Fan Yang, Jiaying Qiu, Lina Wang, Wensi Du, Cong Hua, Zhicheng Xu, Jia Cai, Zhiyong Li, Yiqun Liu, Ling Li, Minwen Zhang, Jing Chen, Lei Zhang, Dongsheng Chen, Weiwen Wang, Shiping Liu, Liang Wu, Wenjun Zeng, Bo Wang, Xiaofeng Wei, Longqi Liu, and Fengzhen Chen. Cdcp: a visualization and analyzing platform for single-cell datasets. Journal of Genetics and Genomics, 49(7):689–692, 2022.

[5] F Alexander Wolf, Philipp Angerer, and Fabian J Theis. Scanpy: large-scale single-cell gene expression data analysis. Genome biology, 19(1):15, 2018.

[6] Zhuxi Jiang, Yin Zheng, Huachun Tan, Bangsheng Tang, and Hanning Zhou. Variational deep embedding: An unsupervised and generative approach to clustering. In Proceedings of the Twenty-Sixth International Joint Conference on Artificial Intelligence, IJCAI-17, pages 1965–1972, 2017.

[7] Lin Yang, Wentao Fan, and Nizar Bouguila. Clustering analysis via deep generative models with mixture models. IEEE Transactions on Neural Networks and Learning Systems, 33(1):340–350, 2020.

[8] Zheng R, Li M, Liang Z, et al SinNLRR: a robust subspace clustering method for cell type detection by non-negative and low-rank representation. Bioinformatics, 35: 3642–50, 2019.

[9] Yan X, Zheng R, Li M. GLOBE: a contrastive learning-based framework for integrating single-cell transcriptome datasets. Brief Bioinform, 23: bbac311, 2022.

[10] Chichi Dai, Yi Jiang, Chenglin Yin, Ran Su, Xiangxiang, Zeng, Quan Zou, Kenta Nakai, and Leyi Wei. scimc: a platform for benchmarking comparison and visualization analysis of scrna-seq data imputation methods. Nucleic Acids Research, 50(9):4877–4899, 2022.
